# Characterization of flexible RNA binding by tandem RNA recognition motifs through integrative ensemble modelling

**DOI:** 10.1093/nar/gkag269

**Published:** 2026-03-31

**Authors:** Cristina K X Nguyen, Laura Esteban-Hofer, Fred F Damberger, Maxim Yulikov, Peter Güntert, Laura Galazzo, Antoine Cléry, Gunnar Jeschke, Frédéric H -T Allain

**Affiliations:** Department of Biology, Institute of Biochemistry, ETH Zürich, Hönggerbergring 64, CH-8093, Switzerland; Department of Chemistry and Applied Biosciences, ETH Zürich, Vladimir-Prelog-Weg 2, CH-8093, Switzerland; Department of Biology, Institute of Biochemistry, ETH Zürich, Hönggerbergring 64, CH-8093, Switzerland; Department of Chemistry and Applied Biosciences, ETH Zürich, Vladimir-Prelog-Weg 2, CH-8093, Switzerland; Department of Chemistry and Applied Biosciences, ETH Zürich, Vladimir-Prelog-Weg 2, CH-8093, Switzerland; Institute of Biophysical Chemistry, Goethe University, Max-von-Laue-Str. 9, 60438, Germany; Department of Chemistry, Tokyo Metropolitan University, 1-1 Minami-Osawa, Hachioji, 192-0397, Japan; Department of Chemistry and Applied Biosciences, ETH Zürich, Vladimir-Prelog-Weg 2, CH-8093, Switzerland; Department of Biology, Institute of Biochemistry, ETH Zürich, Hönggerbergring 64, CH-8093, Switzerland; Department of Chemistry and Applied Biosciences, ETH Zürich, Vladimir-Prelog-Weg 2, CH-8093, Switzerland; Department of Biology, Institute of Biochemistry, ETH Zürich, Hönggerbergring 64, CH-8093, Switzerland

## Abstract

Multi-functional, promiscuous RNA-binding proteins may exhibit substantial disorder in both their free and bound states. The tandem RNA recognition motifs (RRMs) of SRSF1 can serve as a model for such behaviour, as they bind RNA with RRM1 either upstream or downstream from RRM2. Here, we study binding of two short single-stranded RNAs with different directionality to a construct featuring the tandem RRMs and their 30-residue flexible linker. We integrate NMR paramagnetic relaxation enhancement (PRE) and electron paramagnetic resonance (EPR) distance distribution restraints for ensemble modelling. Binding only moderately reduces the occupied conformation space. Ensembles with RRM2 bound downstream or upstream from RRM1 are clearly distinguishable. The system can serve as a testbed for integrative ensemble modelling approaches. Initial conformer pools generated by AlphaFold3 are more compact than those generated by other approaches. AlphaFold3 fails to predict RNA binding of RRM2. Ensembles generated with MMMx using rigid body search to fit distance distributions, followed by selection of a population-weighed subset of conformers consistent with both EPR data and PREs, gave the best fit, and were robust to elimination of individual distance distributions. We also present a less demanding PRE-only approach using multi-state structure calculations with CYANA.

## Graphical Abstract

**Graphical Abstract:**
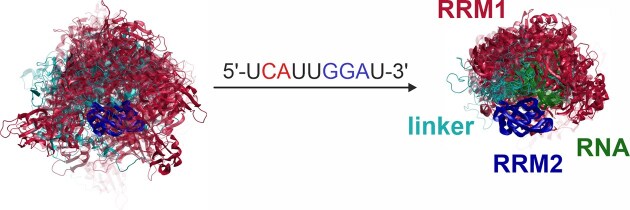

## Introduction

The function of most proteins depends on their binding to small molecules, other proteins, or nucleic acids. Historically, proteins and their binding partners were considered to exhibit a single conformation. This led Emil Fischer to the hypothesis that enzyme and substrate fit each other’s 3D structure like a lock and a key. When evidence mounted that proteins in solution usually adopt an ensemble of conformers and may change conformation upon binding, induced folding was proposed as a binding mechanism [1]. Over the years, clear evidence arose for such folding upon binding [2]. However, it was also observed that disorder may persist after binding [3]. From an evolutionary perspective, disorder is often conserved, which can be explained by the need to balance specificity and adaptability of binding [4]. For proteins involved in RNA processing, this requirement arises because they distinguish between different classes of RNA rather than being specialized to a single RNA with a fixed 3D structure.

One of the ways for achieving RNA-class specificity are tandem RNA-binding domains that bind RNA synergistically. This increases affinity as well as specificity by combining two binding sites on the RNA in a spatially restrained way. Binding becomes more versatile by relying on short binding motifs in the RNA and allowing for some variability in the binding pose. Usually, tandems of RNA recognition motifs (RRMs) bind RNA in a single orientation, i.e. the order of the RRMs is always the same with respect to the 5′-to-3′ direction of the RNA chain [5]. Here, we focus on an exception from this pattern, human Serine Arginine Splicing Factor 1 (SRSF1 or SF2/ASF, UniProt Q07955). SRSF1 is the prototypical member of the phylogenetically conserved SR protein family of gene regulators [6, 7]. This protein family is required for pre-mRNA (pre-messenger RNA) splicing and regulates alternative splicing [8]. In addition, family members are involved in several post-splicing processes, such as mRNA nuclear export, nonsense-mediated decay, and translation [9, 10]. The misregulation of SR protein functions has been related to a wide range of diseases, such as cancer and spinal muscular atrophy [11–13].

**Figure 1.**
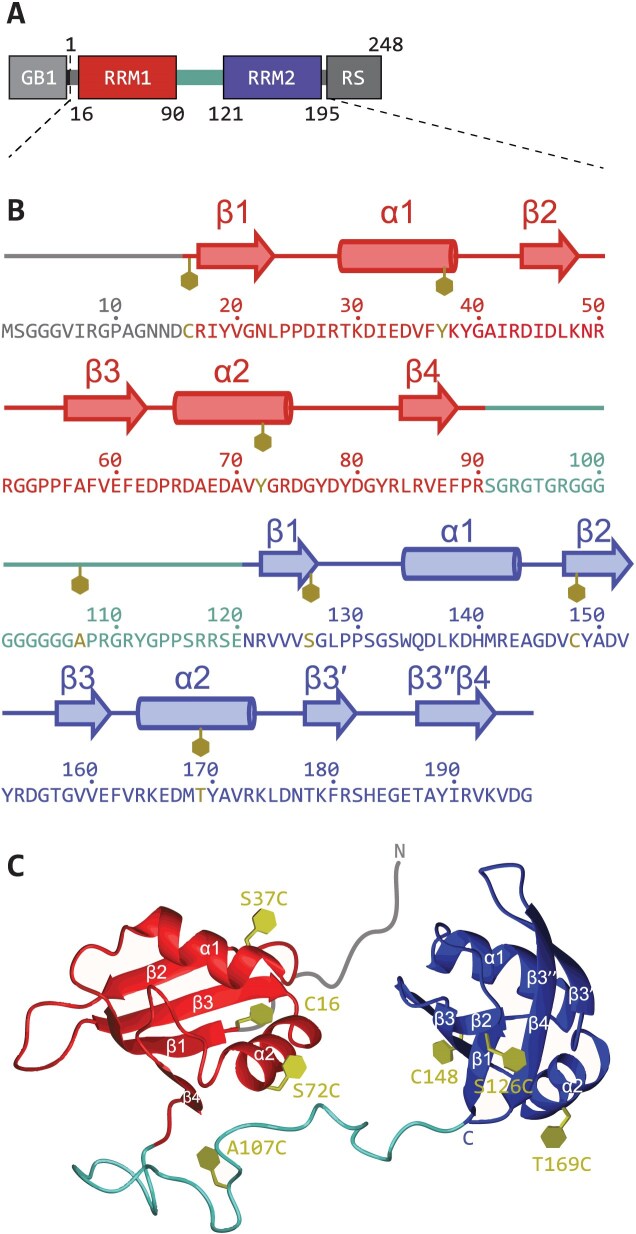
SRSF1 domain boundaries, secondary, and tertiary structure of the SRSF1$_{\Delta \mathrm{RS}}$ construct used in this work. (**A**) The SRSF1 construct contains a GB1 solubility tag (light grey), a (His)6 tag (black), and residues 1–196 of SRSF1 comprising RRM1 (red), a flexible linker (cyan), and RRM2 (blue). The intrinsically disordered RS domain (dark grey) was deleted. (**B**) Secondary structure of RRM1 and RRM2 with positions of spin-labelled sites used indicated by gold hexagons. Residue number positions are indicated by a dot above every tenth residue. (**C**) Tertiary structure of SRSF1$_{\Delta RS}$ with used spin-label positions indicated by gold hexagons. Secondary structure and chain termini are also indicated. RNA-binding sites are located on the back side of the RRM1 $\beta$-sheet, and RRM2 helix $\alpha$1.

SRSF1 embeds the canonical RRM1 (residues 16–90) followed by a pseudo-RRM ($\psi$RRM2 or RRM2, residues 121–195) and a C-terminal RS domain like all other SR proteins (Fig. 1) [9]. Whereas the RRM domains have well-defined folds, the RS domain has a low complexity sequence, embedding a large proportion of serine and arginine residues. Using SELEX, we have recently found that SRSF1 binds equally well ($K_\mathrm{d}$ = 58 nM) to 5′-UCAUUGGAU-3′ (RNA9) and 5′-UGGAUUUUUCAU-3′ (RNA12), where the CA motif recognized by RRM1 is positioned either 4 nucleotides (nt) upstream or 6 nt downstream of the GGA motif recognized by RRM2 [14]. This flexibility in binding pose is probably enabled by a glycine-rich linker of 30 residues (91–120) between the two RRMs, which provides high flexibility [15]. This existence of alternative binding modes is not only of interest for understanding specificity and versatility of SRSF1 in itself but also provides a test case for structural characterization of flexible binding. To this end, we determine ensemble models of free SRSF1, and SRSF1 in complex with RNA9 and RNA12.

Compared to the determination of structures of intrinsically folded regions (IFRs) of proteins with atomic resolution, ensemble modelling [16–22] is less standardized and much less applied [23, 24], although further progress towards generalized approaches has been made recently [25–28]. There is still no consensus on optimum ensemble size, on proof of sufficiency and consistency of sets of experimental restraints, and on assessment of the uncertainty of ensemble models. In particular for proteins such as SRSF1, which feature several IFRs linked by intrinsically disordered regions (IDRs), it may also be difficult to achieve or prove sufficient sampling of conformational space. Most ensemble modelling relies on integration of restraints from different experimental techniques [29], since no single technique covers all length scales of interest.

Here, we use as the main source of information distance distribution restraints for pairs of spin labels, as they can be obtained by the electron paramagnetic resonance (EPR) technique of double electron electron resonance (DEER) [30]. Unlike the mean-value restraints provided by most other techniques, such distribution restraints contain direct information on the width of the ensemble. On the other hand, such distance distribution restraints, which are obtained at cryogenic temperatures (50 K) in the solid state, may be affected by perturbation of the conformer distribution due to the introduced labels, and are sparse because of the large effort required for determining each single distribution restraint. Their relatively low resolution stemming from a distribution of rotameric states of the spin label sidechain [31] and their lower distance limit of about 15 Å [32] limit information on local details. Therefore, we complement the DEER restraints with NMR paramagnetic relaxation enhancement (PRE) restraints which are sensitive to distances in the range of $\approx 12-25$ Å [33].

For efficiency reasons, we rely on an approach where a large pool of conformers sampled in a first step is reweighed and contracted in a second step. Thus, the quality of the fit to experimental data critically depends on sampling quality of the initial conformer pool [34]. Therefore, we compute initial pools by three different approaches, the torsion-angle dynamics algorithm CYANA [35] typically used in high-resolution NMR structure determination, the RigiFlex approach that utilizes distance distribution restraints already to improve efficiency of initial conformer sampling [36], and AlphaFold3 [37] ensemble modelling. The latter might improve sampling by evolutionary information in its pairformer module and by information on local structure in its diffusion module, which was trained on experimental protein structures. We compare the outcomes from the different initial pools. Further, we validate the restraint sets by jackknife resampling with the RigiFlex approach and generate an enhanced-sampling superensemble [38]. We also investigate a slimmer alternative method relying only on the PRE restraints, accounting for the dynamic averaging of PREs by using a multi-state CYANA calculation. Finally, we analyse how the conformation ensemble changes upon binding of RNA9 and RNA12 to SRSF1.

## Materials and methods

All reagents were purchased from Merck unless otherwise specified.

### Expression and purification of the recombinant proteins

The coding sequences for SRSF1 RRM1 (amino acids 1–90 of SRSF1), RRM2 (amino acids 107–203 of SRSF1), and SRSF1$_{\Delta \mathrm{RS}}$ (amino acids 1–196 of SRSF1) were cloned into the bacterial vector pET24 [14, 39]. All the recombinant proteins were fused to an N-terminal GB1 solubility tag followed by a 6$\times$His tag for the purification. In addition, to increase the solubility, Y37S and Y72S point mutations were performed. Due to the low solubility of the full-length protein limiting NMR measurements, we did not include the RS domain in our constructs. All the recombinant proteins were overexpressed at 37°C in *Escherichia coli* BL21 (DE3) codon plus cells in LB-broth medium (BD Difco) for non-isotopically labelled protein, or in M9 minimal medium supplemented with 1 g/l $^{15}\mathrm{NH}_{4}\mathrm{Cl}$ (Merck or Cambridge Isotope Laboratories) and 2 g/l $^{13}$C$_6$-glucose for $^{13}$C,$^{15}$N- or 4 g/l glucose for $^{15}$N isotopically labelled protein, respectively. In addition, 50 µg/ml kanamycin and 50 µg/ml chloramphenicol were added to the media. The cell cultures were collected by centrifugation at 4000 rpm and 4°C for 15–20 min. The cell pellet was then resuspended in 20 ml of lysis buffer (50 mM $\mathrm{Na}_{2}\mathrm{HPO}_{4}$, 1 M NaCl, pH 8) and lysed using a microfluidizer. The resulting cell lysate was clarified by centrifugation at 17 000 rpm and 4°C for 45 min, and the protein of interest was purified using nickel affinity chromatography (QIAGEN). The protein solution was loaded onto a Nickel column and washed first with wash buffer A (20 mM $\mathrm{Na}_{2}\mathrm{HPO}_{4}$, 3 M NaCl, pH 8) to remove nucleic acids and then with 15–20 ml of wash buffer B (50 mM $\mathrm{Na}_{2}\mathrm{HPO}_{4}$, 50 mM L-Arg, 50 mM L-Glu, 1 M NaCl, and 40 mM imidazole, pH 8) to remove nonspecifically bound proteins. The protein was then eluted with 10–15 ml of elution buffer (50 mM $\mathrm{Na}_{2}\mathrm{HPO}_{4}$, 1 M NaCl, and 200 mM imidazole, pH 8) and dialyzed in dialysis buffer (20 mM NaPi, 50 mM L-Arg, 50 mM L-Glu, and 3 mM DTT (Dithiothreitol) at pH 6). The purity of the protein sample was checked by running it on a 12% SDS gel. The recombinant proteins were concentrated using a 10-kDa molecular mass cutoff Centricon device (Vivascience). Finally, the protein samples were stored at −20°C until needed for further use.

### Site-directed spin labelling of the protein samples

The reducing agent present in the buffer (3 mM DTT) was removed with a desalting column filled with Sephadex G-25 resin (PD10, PD MidiTrap, or PD MiniTrap column, Cytiva). Subsequently, the protein sample was incubated overnight at room temperature (RT) with a ten-fold excess of either MTSL [(1-oxyl-2,2,5,5-tetramethylpyrroline-3-methyl) methanethiosulfonate; Toronto Research Chemicals] or Gd(III)-maleimido-DOTA (1,4,7,10-tetraazacyclododecane-1,4,7-tris-acetic acid-10-maleimiodethylacetamide). Following this, the unreacted spin label was removed using a PD10 column (GE Healthcare) and the labelled protein was subsequently concentrated up to a maximum concentration of 200 µM via a 10-kDa molecular mass cutoff Centricon device (Vivascience). The labelling efficiency was determined using continuous-wave (CW) EPR spectroscopy for MTSL and Mass Spectrometry for Gd(III). For DEER samples, the final step was carried out in EPR buffer (20 mM NaPi, 50 mM L-Arg, 50 mM L-Glu, pH 6 with 10% glycerol), while for the PRE samples, glycerol was omitted. Labelling yields were 120%–150% for double mutants, corresponding to 60 to 75% for individual labelling sites.

### Site-directed spin labelling of RNA

Site-directed spin labelling of RNA required the introduction of a thiophosphate at the 5′-terminus and the deprotection of a 3′-end thiol-modified RNA. Labelling was quantitative.

#### Introducing the 5′-end modifier

The unmodified RNAs, RNA9 (5′-UCAUUGGAU-3′) and RNA12 (5′-UGGAUUUUUCAU-3′) (Dharmacon) were deprotected by incubation with deprotection buffer (100 mM acetic acid, adjusted to pH 3.8 with tetramethylethylenediamine) for 30 min at 60°C. The RNAs were subsequently lyophilized overnight and resuspended in 100 µl water to a final RNA concentration of approximately 1.5 mM. The RNAs were then thiophosphorylated at the 5′-end by mixing 30 nmol RNA, 15 µl of 10× PNK buffer (NEB), 15 µl of T4 PNK (NEB), 150 nmol ATP-$\gamma$-S, and addition of milli-Q water to a total volume of 150 µl. After overnight incubation at 37°C, the RNA mixture was desalted by addition of 16.5 µl of a 3 M sodium acetate solution at pH 5.2, 50 nmol MgCl_2_ (NEB), and 450 µl of ethanol. This mixture was kept on ice for 2 h, and the precipitated RNA was then separated from the rest of the solution by centrifugation at 10 000 rcf for 1 h at 4°C. Next, the pellet was washed with 300 µl of cold ethanol. After an additional centrifugation step for 20 min, the supernatant was discarded, and the pellet dried for 2 min at 70°C before resuspending it in 500 µl of a 100 mM $\mathrm{K}_{2}\mathrm{HPO}_{4}$ buffer at pH 8.

#### Deprotection of the 3′-end thiol-modified RNAs

The RNAs, RNA9 and RNA12 containing a 3′-end thiol modifier (C3 S-S) were purchased from Integrated DNA Technologies, which were sythesized with the sulfur atom protected by an S=S bond. 30 nmol of each RNA were deprotected by incubation in a 100 mM DTT, 20 mM $\mathrm{Na}_{2}\mathrm{HPO}_{4}$, pH = 8.4 buffer for approximately 1 h at room temperature. The RNA was then desalted by the same ethanol precipitation protocol as specified above.

#### Introducing modifiers at both RNA termini

Spin labelling the RNA at both termini required the introduction of two modifiers within one RNA strand. For this, the 3′-end thiol-modified RNAs were phosphorylated at the 5′-end with T4 polynucleotide kinase (New England Biolabs) prior to reduction with DTT. The RNAs were then precipitated and resuspended in a 100 mM $\mathrm{K}_{2}\mathrm{HPO}_{4}$, pH 8 buffer.

#### RNA spin-labelling

The modified and deprotected RNAs were spin labelled by addition of 3-(2-Iodoacetamido)-PROXYL in high molar excess (50$\times$ per labelling site) and incubation for 2–3 days at 4°C. The unreacted spin label was removed by buffer exchange into $\mathrm{H}_{2}\mathrm{O}$ with HiTrap Desalting columns with Sephadex G-25 resin (Cytiva) on an Aekta purifier system (Cytiva). The elution of the RNA was monitored by the absorbance at 260 and 280 nm. Fractions close to the peak maximum were pooled and then lyophilized. The RNA pellet was resuspended in NMR buffer with $\mathrm{D}_{2}\mathrm{O}$ with a final concentration of 300 to 400 µM. The labelling efficiency was determined by CW EPR experiments.

### NMR experiments

All NMR experiments were conducted with the sample solubilized in NMR buffer comprising of 20 mM NaPi, 50 mM L-Arg, 50 mM L-Glu, and 3 mM DTT at pH 6. However, for PRE experiments, samples were prepared in the absence of DTT to prevent spin-label reduction. In the case of protein–RNA complexes, a 1:1.2 ratio of protein and RNA was mixed to form the complex. NMR spectra were acquired at 313 K on Bruker AVIII-500 MHz, AVIII-600 MHz, AVIII-700 MHz, and Avance-900 MHz spectrometers equipped with a cryoprobe. TopSpin 3.x or 4.x (Bruker) was used to process the spectra, and their analysis was performed using DynamicsCenter (Bruker), Sparky [40], and CARA [41].

#### Chemical shift differences analysis

$^{1}$ H-$^{15}$N heteronuclear single quantum coherence (HSQC) experiments were performed to characterize and compare $^{15}$N isotope-labelled samples of SRSF1 RRM1, SRSF1 RRM2, and SRSF1$_{\Delta \mathrm{RS}}$. Backbone assignments of the isolated RRMs in the free state and in complex with RNA were previously performed in a different buffer [14, 39], and it was possible to transfer these assignments to the NMR buffer conditions used here by similarity of the $^{15}$N,$^{1}$H-HSQC spectra. The new assignments were confirmed using triple resonance spectra of the $^{13}$C,$^{15}$N-labelled proteins. To account for differences between the spectra obtained in different conditions, peak positions were adapted for each set of spectra. Chemical shift differences between the different constructs were then calculated using the formula:


(1) \begin{eqnarray*}
\delta \mathrm{CS} = \sqrt{\left(\delta \mathrm{HN}\right)^2 + \left(\delta \mathrm{N}/6.51\right)^2}
\end{eqnarray*}

where $\delta$HN and $\delta$N are the chemical shift differences of the amide proton and the nitrogen atom, respectively. Secondary structure propensities were computed from chemical shifts by TALOS-N [42].

#### $^{15}\mathrm{N}-\lbrace ^{1}\mathrm{H}\rbrace$ Heteronuclear NOE experiments

Heteronuclear $^{15}$N-$\lbrace ^{1}\mathrm{H}\rbrace$ nuclear Overhauser effect (NOE) experiments were performed to characterize the subnanosecond timescale dynamics of the backbone of the SRSF1$_{\Delta \mathrm{RS}}$ construct. The experiment was conducted at a proton frequency of 700 MHz with a relaxation delay of 2 and a 3.5 s saturation period in the saturation experiment and a corresponding delay in the reference experiment. The NOE values were then determined by calculating the ratios of peak intensities in the saturated spectrum to those in the unsaturated spectrum.

#### Protein dynamics study: $^{15}$N spin-relaxation experiments

To investigate the correlation time of the isolated RRMs and the RRMs in SRSF1$_{\Delta \mathrm{RS}}$ (free state and in complex with RNA), $^{15}$N longitudinal relaxation times *T*$_1$ and transverse relaxation times *T*$_2$ were recorded at a $^{1}$H frequency of 600 MHz using established and previously described protocols [43–45]. For *T*$_1$, seven relaxation delays plus one repetition were measured in an interleaved manner using *T*$_1$ relaxation delays of 0.01/0.15($\times$2)/0.3/0.6/1/1.5 and 2 s. For *T*$_2$, seven relaxation delays plus one repetition were measured in an interleaved manner using *T*$_2$ relaxation delays of 0 /17/34/68($\times$2)/102/204 and 340 ms. Relaxation times in both *T*$_1$ and *T*$_2$ relaxation experiments were interleaved such that long relaxation times followed short ones. Peak lists obtained from backbone resonance assignments previously [14, 39] were carefully adjusted to match the relaxation data, and intensities of individual well resolved peaks were extracted and fitted to an exponential decay using DynamicsCenter (Bruker). Errors in the fit were used to estimate the error of the determined relaxation time based on Monte Carlo simulation within DynamicsCenter. The correlation time was then calculated from the *T*$_1$ and *T*$_2$ times using the equation:


(2) \begin{eqnarray*}
\tau _\mathrm{c} = \frac{1}{4\pi \nu _\mathrm{N}}\sqrt{6\frac{T_1}{T_2}-7}
\end{eqnarray*}

where $\nu _\mathrm{N}$ is the $^{15}$N resonance frequency (60.82 MHz) [46]. Errors for $\tau _\mathrm{c}$ were evaluated by error propagation with the above formula using the errors in the *T*$_1$ and *T*$_2$ values.

#### PRE experiments

The correct folding of each mutant was verified by recording $^{1}$H-$^{15}$N HSQC experiments in the presence and absence of a diamagnetic version of the spin probe, and the paramagnetic sample labelling was confirmed using CW EPR experiments. PREs were obtained from the difference in $^{1}$HN-*T*$_2$ relaxation rates of paramagnetic and diamagnetic samples of the same preparation. Measurements were carried out for all the six single-cysteine mutants labelled with diamagnetic and paramagnetic probes, both in the free form and in complex with RNA9 or RNA12. $^{1}$H$^\mathrm{N}$-*T*$_2$ relaxation experiment consisted of a $^{15}$N,$^{1}$H-HSQC modified to use a variable delay for the first INEPT transfer while maintaining the optimal scalar coupling evolution as described previously [47]. The $^{1}$H inversion pulse in the INEPT transfers was replaced with an amide selective inversion pulse. For the paramagnetic samples, seven relaxation delays plus one repetition were measured in an interleaved manner (6/7/8/10($\times$2))/12/14 and 20 ms). For the diamagnetic samples, seven relaxation delays plus one repetition were measured in an interleaved manner (6/8/10($\times$2))/12/14/16 and 20 ms). Each $^{15}$N,$^1$H-HSQC of the relaxation series was measured with 64 scans. Peak lists were carefully adjusted to match peak positions, and intensities of individual peaks were extracted and fitted to an exponential decay using DynamicsCenter (Bruker). Errors in T$_2$ relaxation times were estimated based on the error in the fit using Monte Carlo simulation within DynamicsCenter. The PRE rate constants were determined by subtracting the transverse relaxation rates $R_{2,\mathrm{dia}}$ of the diamagnetic samples from the $R_{2,\mathrm{para}}$ rates of the paramagnetic samples. This difference between the two rates is also represented as $\Gamma _2 = R_{2,\mathrm{para}} - R_{2,\mathrm{dia}}$. In instances where amide peaks were excessively broadened and not detectable during the acquisition of *R*$_2,\mathrm{para}$, we assigned a value of $\Gamma _2$ = 170 Hz. To derive dynamically averaged distance restraints for the multi-state CYANA calculation, the distances from the unpaired electron to individual amide $^{1}\mathrm{H}$ atoms were calculated using the formula:


(3) \begin{eqnarray*}
r = \left[ \frac{K}{\Gamma _2}\left(4 \tau _\mathrm{c} + \frac{3 \tau _\mathrm{c}}{1+\omega _\mathrm{H}^2 \tau _\mathrm{c}^2} \right)\right]^{1/6}
\end{eqnarray*}

where $K = 1.23\cdot 10^{-23} \ \mathrm{cm}^6 \ \mathrm{s}^{-2}$ [48] is the magnetic susceptibility difference between the paramagnetic center and the surrounding medium, $\Gamma _2$ is the transverse relaxation rate enhancement of the nucleus, $\tau _\mathrm{c}$ is the correlation time of the amide $^{15}$N, and $\omega _\mathrm{H}$ is the Larmor frequency of the $^{1}$H nucleus. We did not consider uncertainty of $K$ in error propagation.

Each distance determined by a PRE was converted into upper and lower distance restraints where the upper distance limit (upl) corresponded to the distance r plus the error in the distance, and the lower distance limit (lol) was defined as the distance minus the error in the distance. The error in the distance was determined by error propagation of the above formula using the errors in the PRE relaxation rate ($\Gamma _2$) and $\tau _\mathrm{c}$. Amide signals which disappeared in $^{15}$N,$^{1}$H-HSQC spectrum of the paramagnetic sample were interpreted to be closer than 12 Å to the spin label and only upl restraints were defined. No distance restraints were defined in cases where the error was $ > 50$% of the distance itself, as it was deemed that such distances were unreliable. For the CYANA calculations, the inter-RRM distance restraints were defined between the DUMMY atom Q8 representing the centre of the spin-label cloud of the labelled residue (see Modelling with multi-state CYANA) and the NH atom of the residue showing a PRE.

To obtain the PRE ratios ($I_\mathrm{para}/I_\mathrm{dia}$), the integrated peaks of the $^{15}$N,$^{1}$H-HSQC plane obtained with the shortest relaxation time (6 ms) were normalized by the integral of the reference peak (chosen as the most intense peak in the GB1-tag). The PRE ratio $I_\mathrm{para}/I_\mathrm{dia}$ was then calculated with the normalized integrals of the paramagnetic $I_\mathrm{para}$ and the diamagnetic sample $I_\mathrm{dia}$.

### EPR experiments

#### Continuous-wave EPR measurements

CW EPR spectra were acquired at room temperature on a Bruker Elexsys E500 spectrometer equipped with a super high-Q resonator ER4122SHQ at X band frequencies ($\approx 9.5$ GHz). Protein samples (20 µl) were filled into a microcapillary tube (BLAUBRAND). All measurements were recorded with 100 kHz field modulation and 0.15 mT modulation amplitude. The labelling efficiency was calculated by double integration of the spectrum and comparison with a sample of known concentration.

#### DEER sample preparation and measurement

All samples were measured in a deuterated matrix achieved by repeatedly concentrating the samples using 10 kDa molecular weight cutoff filter units (Amicon Ultra-0.5) and replacing the volume by EPR buffer prepared with $\mathrm{D}_{2}\mathrm{O}$. The protein–RNA complex was formed by mixing protein and RNA at the appropriate ratios, and incubation at 37°C for 10 min. The protein–RNA ratio was 1:1.2 for the intra-protein distance restraints, 1:1 for the protein–RNA measurements, and 1.2:1 for the intra-RNA restraints. D8-glycerol was added to yield a final cryoprotectant concentration of 50%. Final protein concentration varied from approximately 10–35 µM depending on the concentration of the stock. Samples were shock-frozen in liquid nitrogen.

##### Nitroxide-nitroxide DEER measurements

Pulsed EPR measurements were performed on a homebuilt Q-band spectrometer ($\approx 34$ GHz) [32]. A sample tube loaded with 35 µl sample was shock-frozen in liquid nitrogen and subsequently inserted into a homebuilt Q-band resonator for 3 mm outer-diameter sample tubes [32]. Nitroxide-nitroxide DEER experiments were acquired at a temperature of 50 K, achieved by liquid-helium cooling and controlled by a He flow cryostat (ER 4118CF, Oxford Instruments) and a temperature control unit (ITC 503, Oxford Instruments). An echo-detected field-swept EPR spectrum was acquired using a Hahn-echo sequence. The pulse sequence for the four-pulse DEER experiment was $\pi /2_\mathrm{obs}-\tau _1-\pi _\mathrm{obs}-t_1-\pi _\mathrm{pump}-(\tau _1 + \tau _2 - t_1)-\pi _\mathrm{obs}-\tau _2$. The pump pulse was applied on the spectral maximum and the observer pulses were applied at a frequency offset of 100 MHz. All measurements were acquired with a 16 ns pump pulse, and 12 ns or 16 ns observer pulses, a pulse delay $\tau _1$ of 400 ns, an initial delay $t_1$ of 280 ns, and eight-step nuclear modulation averaging with an averaging time step of 16 ns. The pulse delay $\tau _2$ and the time scale increment were adjusted according to the expected distance.

##### Gd(III)-nitroxide DEER measurements

The Gd(III)-nitroxide DEER experiments were performed with the following adjustments. The orthogonal measurements were acquired at a temperature of 10 K. The pump pulse was applied on the spectral maximum of the nitroxide spectrum and the observer pulses at the maximum of the Gd(III) spectrum, which corresponds to a 300 MHz frequency offset. We employed 12 ns pulses and a $\pi /2$ pump pulse, as previously optimized by Garbuio *et al.* [49]. Otherwise measurements were performed as for nitroxide labels.

#### DEER data analysis

DEER traces were analysed with a Matlab-based DeerLab version (release 0.9.2) [50]. The primary DEER data were submitted to a zero-time and phase correction and cropped to remove the “2+1” artefact at the end of the trace. Distance distributions were obtained by a one-step analysis, in which the background and the non-parametric distribution are fitted simultaneously. A stretched exponential function was assumed in the background correction, and the regularization parameter was selected by either the Bayesian information criterion, the residual method, or the L-curve minimum-radius method. For all fitted signals, distributions, and parameters, bootstrapped confidence intervals were estimated from 1000 bootstrap samples drawn from a Gaussian distribution based on the noise in the measurements. Distance restraints in RigiFlex are specified by a mean distance and standard deviation. Hence, all the distributions were overlaid by a Gaussian. This was done by either analysing the primary DEER data by a single-Gaussian or by least-square fit of the non-parametric distance distribution by a Gaussian function of the form:


(4) \begin{eqnarray*}
P(r) = A \exp {\left[-\frac{\left( r - \left\langle r \right\rangle \right)^2}{2\sigma ^2}\right]}
\end{eqnarray*}

where $A$ is a normalization factor, $\left\langle r \right\rangle$ the mean distance, and $\sigma$ the standard deviation. The analyses of both approaches were compared, and the Gaussian that best described the non-parametric distance distribution was selected as distance restraint. In a few cases, when both the time-domain and the distance-domain Gaussian fits represented the underlying distributions poorly, the overlay was optimized manually. Note that Gaussian restraints were used only for generation of the RigiFlex initial pool. Ensemble reweighing was performed by fitting the non-parametric distance distributions.

### Ensemble modelling

Pools of about $1200 - 2000$ conformers were generated by the RigiFlex approach in MMMx [36], AlphaFold3 [37], and CYANA [35]. The models were generated by ensemble reweighing in MMMx [36] while discarding all conformers with weights smaller than 1% of the largest weight. DEER distance distributions were backcalculated with MMMx [36] based on a rotamer library approach for the spin label [31]. PREs were backcalculated with the MMMx implementation of the algorithm used in DEER-PREdict [51], which is in turn based on the same rotamer library approach and on a simplified dynamics model that accounts for flexibility of the label [52]. We assumed a correlation time for internal motion of 0.5 ns and the experimentally determined rotational correlation times for the protein. Backcalculated PREs are obtained from the weighted mean of PREs calculated for individual conformers.

#### Conformer pools by RigiFlex

The conformer pools were generated on the ETH Euler cluster with the software package MMMx [36] (version from 21 December 2021 with dependencies on YASARA [53] and SCWRL4.11 [54]). The construct included residues 16–195. The rigid-body templates were prepared prior to the ensemble generation using MMM [26]. For SRSF1$_{\Delta \mathrm{RS}}$ in the free form, PDB structures, 1X4A and 2O3D [55], were used for RRM1 (residues 16–89) and RRM2 (residues 121–195), respectively. We introduced the RRM1 mutations Y37S and Y72S *in silico* by MMM. The rigid bodies of RRM1 in complex with CA and RRM2 in complex with GGA were derived from the PDB structures: 6HPJ2 [14] and 2M8D [39]. The rigid bodies were first arranged relative to each other via RRM–RRM Gaussian distance restraints using the Rigi module (see Supplementary Fig. S1A). Based on the number of successfully generated conformers in smaller trials, we started with a different number of rigid body arrangements for free SRSF1$_{\Delta \mathrm{RS}}$ (2000), SRSF1$_{\Delta \mathrm{RS}}$ in complex with RNA9 (6000), and SRSF1$_{\Delta \mathrm{RS}}$ in complex with RNA12 (8000), selected by hierarchical clustering from 50 000 initial arrangements (or in the case of the complex with RNA12 from 150 000 initial arrangements). The larger size of the rigid-body arrangement ensembles for the complexes compensates for losses in the FlexRNA step that occur because for some of the rigid-body arrangements, generation of the RNA linker between the binding motifs fails. The RNA linkers were generated by FlexRNA [26] which is based on random sampling of backbone conformations from a pseudo-torsional angle library for RNA [56]. For the SRSF1$_{\Delta \mathrm{RS}}$–RNA complexes, the two RNA-binding motifs were connected in the FlexRNA module (Supplementary Fig. S1B) by 5′-UU-3′ or 5′-UUUUU-3′ for the complexes with RNA9 and RNA12, respectively. The 5′-end uracil was inserted using the RRM-U1 distance distribution restraints, and, subsequently the 3′-end uracil was introduced using the U1-U9 or U1-U12 distance distribution restraint. Lastly, the RRMs were connected by the inter-domain linker using the Flex module (Supplementary Fig. S1C). This step considered RRM-linker restraints for free SRSF1$_{\Delta \mathrm{RS}}$, and RRM-linker and RNA-linker restraints for the protein–RNA complexes. With exception of the Flex step, which employs rejection sampling of non-parametric distance distributions, the remaining modules are based on distance restraints specified by mean distances and standard deviations. Flex can consider secondary structure propensities by biased sampling from the residue-specific Ramachandran plots. As chemical shift analysis did not indicate significant secondary structure propensities in the linker, we refrained from such biasing. Conformers generated by the RigiFlex pipeline were refined with YASARA Structure [53]. The size of RigiFlex conformer pools is reported in Table 1.

**Table 1.** Overview of ensemble models, their mean overlap deficiency $1-\bar{o}$, mean earth mover’s distance (EMD), mean $\chi ^2$ of all PRE restraints $\chi ^2_\mathrm{PRE}$, loss of merit $L$, radius of gyration $R_\mathrm{g}$, and disorder parameter $\delta$

	Pool									
System	Method	Size	Fitted	Conformers	$1-\bar{o}$	EMD (Å)	$\chi ^2_\mathrm{PRE}$	$L$	$R_\mathrm{g}$ (Å)	$\delta$
SRSF1$_{\Delta \mathrm{RS}}$ free	CYANA	2000	None	2000	0.335	16.9	40.947	n.a.	31.4	1.022
SRSF1$_{\Delta \mathrm{RS}}$ free	CYANA	2000	DEER	68	0.076	0.6	39.859	n.a.	20.8	0.636
SRSF1$_{\Delta \mathrm{RS}}$ free	CYANA	2000	PRE	29	0.344	10.6	5.456	n.a.	25.8	0.548
SRSF1$_{\Delta \mathrm{RS}}$ free	CYANA	2000	All	69	0.080	0.7	6.348	0.080	20.7	0.591
SRSF1$_{\Delta \mathrm{RS}}$ free	CYANA MS$^{1}$	2000	PRE	140	0.223	5.4	21.736	n.a.	18.6	0.473
SRSF1$_{\Delta \mathrm{RS}}$ free	AF3	2000	None	2000	0.285	5.8	9.396	n.a.	19.7	0.355
SRSF1$_{\Delta \mathrm{RS}}$ free	AF3	2000	All	31	0.122	1.3	6.544	0.053	19.7	0.427
SRSF1$_{\Delta \mathrm{RS}}$ free	AF3$^{2}$	2000	All	18	0.186	2.4	6.403	0.023	19.7	0.388
SRSF1$_{\Delta \mathrm{RS}}$ free	RigiFlex	1236	None	1236	0.170	3.3	6.656	n.a.	21.9	0.491
SRSF1$_{\Delta \mathrm{RS}}$ free	RigiFlex	1236	PRE	56	0.240	5.1	5.014	n.a.	20.5	0.481
SRSF1$_{\Delta \mathrm{RS}}$ free	RigiFlex	1236	All	55	0.080	0.8	5.699	0.047	21.1	0.487
SRSF1$_{\Delta \mathrm{RS}}$ free	RigiFlex$^{3}$	465	All	89	0.068	0.7	5.648	0.076	$20.8 \pm 0.5$	$0.486 \pm 0.020$
SRSF1$_{\Delta \mathrm{RS}}$ RNA9	AF3	2000	None	2000	0.246	4.7	7.388	n.a.	19.5	0.338
SRSF1$_{\Delta \mathrm{RS}}$ RNA9	AF3	2000	All	38	0.137	2.0	6.711	0.050	20.4	0.369
SRSF1$_{\Delta \mathrm{RS}}$ RNA9	RigiFlex	1140	None	1140	0.192	3.7	7.278	n.a.	22.8	0.314
SRSF1$_{\Delta \mathrm{RS}}$ RNA9	RigiFlex	1140	PRE	11	0.375	6.6	4.952	n.a.	21.8	0.366
SRSF1$_{\Delta \mathrm{RS}}$ RNA9	RigiFlex	1140	All	48	0.104	1.5	6.477	0.032	22.0	0.363
SRSF1$_{\Delta \mathrm{RS}}$ RNA9	RigiFlex$^{4}$	640	All	63	0.080	1.2	6.257	0.036	$22.1 \pm 0.4$	$0.380 \pm 0.020$
SRSF1$_{\Delta \mathrm{RS}}$ RNA12	AF3	2000	None	2000	0.387	8.1	10.470	n.a.	19.5	0.338
SRSF1$_{\Delta \mathrm{RS}}$ RNA12	AF3	2000	All	29	0.211	3.4	4.418	0.052	19.6	0.403
SRSF1$_{\Delta \mathrm{RS}}$ RNA12	RigiFlex	1242	None	1242	0.173	3.9	6.930	n.a.	22.1	0.423
SRSF1$_{\Delta \mathrm{RS}}$ RNA12	RigiFlex	1242	PRE	26	0.313	6.7	3.957	n.a.	20.6	0.449
SRSF1$_{\Delta \mathrm{RS}}$ RNA12	RigiFlex	1242	All	59	0.099	1.6	4.269	0.065	21.5	0.448
SRSF1$_{\Delta \mathrm{RS}}$ RNA12	RigiFlex$^{5}$	598	All	76	0.090	1.4	4.179	0.066	$21.5 \pm 0.5$	$0.408 \pm 0.028$

$^{1}$ CYANA multi-state ensemble.

$^{2}$ Construct without GB1.

$^{3}$ Pool derived from RigiFlex DEER/PRE ensemble and the superensemble obtained by jackknife resampling. The total size of the primary underlying pool is 4309 conformers. Error estimates were obtained by analysis of the ensemble of ensembles.

$^{4}$ Pool derived from RigiFlex DEER/PRE ensemble and the superensemble obtained by jackknife resampling. The total size of the primary underlying pool is 5596 conformers. Error estimates were obtained by analysis of the ensemble of ensembles.

$^{5}$ Pool derived from RigiFlex DEER/PRE ensemble and the superensemble obtained by jackknife resampling. The total size of the primary underlying pool is 3881 conformers. Error estimates were obtained by analysis of the ensemble of ensembles.

#### Conformer pools by AlphaFold3

Pools of 2000 conformers were generated at the AlphaFold3 [37] server by running 400 jobs each for SRSF1$_{\Delta \mathrm{RS}}$ and the two RNA complexes with different seed values. The protein sequence included the full construct used in the experiments, including the GB1 solubility tag. After downloading the results, the conformers were processed by MMMx [36] to constructs comprising residues 15–196 and the RNA, if present. Unlike RigiFlex and CYANA models, AlphaFold3 models do not contain protons. For PRE prediction with MMMx, the coordinates of NH protons were reconstructed from backbone coordinates of the residue under study and the previous residue in the sequence as implemented in MMMx.

#### Conformer pool by CYANA

All CYANA computations were performed with version 3.98.15. For free SRSF1$_{\Delta \mathrm{RS}}$, a pool of 2000 conformers was generated by CYANA [35]. The construct comprising residues 1–196 was computed using restraints derived from NOEs defined in the structures of the individual RRMs (PDB codes: 1X4A and 2O3D). After minimization, the dihedral angles for the RRM domains were fixed and ensembles were generated by calculating 2000 conformers with randomized linkers.

#### Ensemble reweighing

Ensemble reweighing for all conformer pools was performed with MMMx as described in [36], except here, the earth mover’s distance (EMD) was used instead of overlap deficiency as the figure of merit for fitting distance distributions (see Supplementary Information S1.1, Supplementary Fig. S1D). We implemented this change because the EMD is a proper metric. Briefly, distance distributions for each conformer are predicted by a rotamer library approach [31] while PRE rates for all residues in each conformer were predicted by the approach described in [51] and based on [33]. Backcomputed values were clipped to a maximum of 170 s$^{-1}$. The ensemble prediction for a given vector of conformer weights is a linear combination of the individual conformer predictions with the weights $w_c$ as coefficients. For a given set of conformers, the minimum EMD ($\mathrm{EMD}_\mathrm{min}$) for distance distributions and the minimum $\chi ^2$ value for PRE ($\chi ^2_\mathrm{PRE,min}$) are separately determined by global fitting of the weights. This is followed by another round of global weight fitting where both restraint sets are balanced by minimizing loss of merit $L$ defined by:


(5) \begin{eqnarray*}
L = \frac{1}{2} \left( \frac{\mathrm{EMD}}{\mathrm{EMD}_\mathrm{min}} + \frac{\chi ^2_\mathrm{PRE}}{\chi ^2_\mathrm{PRE,min}} \right) - 1 \ .
\end{eqnarray*}

If $L$ deviates substantially from zero, this indicates inconsistency of the two restraint sets.

#### Jackknife resampling and superensemble generation

Because of the need to introduce two spin labels per restraint and limited availability of suitable labelling sites, distance distribution restraints are sparse. This sparsity is compensated to some extent by the intrinsically low resolution of a structure that corresponds to a broad ensemble of conformers. The extent of disorder is not known beforehand and sufficiency of the restraint set should thus be assessed.

Another potential pitfall arises from the influence of the spin labels on protein conformation, which may or may not be relevant at the resolution of the ensemble. If each labelling site is present in some but not all restraints, substantial perturbations of conformation by the labels would cause inconsistency in the restraint sets.

Jackknife resampling allows for an assessment of both the sufficiency and consistency of a set of distance distribution restraints [38]. Given $R$ distance distribution restraints, we compute $R$ validation ensembles by running the whole RigiFlex modelling and ensemble reweighing pipeline with $R-1$ restraints, omitting one of the restraints per ensemble. From this ensemble, we backcalculate the distance distribution for the omitted restraint and compare it to the experimental distribution. In addition, we compare each of the validation ensembles to the ensemble obtained with all restraints.

This approach provides a new larger pool of conformers by combining the original ensemble and all validation ensembles. This superensemble pool is expected to sample the populated part of conformation space more densely. By applying another round of ensemble reweighing on this pool, we obtain the final RigiFlex ensemble [38].

The validation ensembles from jackknife resampling are further used to derive uncertainty estimates. To this end, we computed the radius of gyration $R_\mathrm{g}$ and a dimensionless disorder parameter $\delta = R_\mathrm{g,ACS}/R_\mathrm{g}$ for all validation ensembles, where $R_\mathrm{g,ACS}$ is the radius of gyration of the ensemble in abstract conformer space [57]. We consider twice the standard deviation of these values as an estimate for the 95% confidence interval of these quantities.

#### CYANA multi-state modelling

As an alternative to the methods described thus far, which rely on EPR derived distance distributions obtained from a series of pairwise spin-labelled samples we also explored a less demanding experimental procedure using only PREs derived from singly spin-labelled samples. In this approach, the PREs were converted to upper and lower distance limits, and CYANA was used to calculate structures compatible with these distance restraints. Because SRSF1$_\Delta \mathrm{RS}$ is likely to be highly dynamic, the measured PREs are dynamically averaged and generally do not correspond to a single distance as would be the case for a rigid structure. Both the spin label and the protein visit different conformations. The spin label was modelled by determining the average position of the center of the unpaired electron relative to the domain it was attached to. In order to account for the different conformations of the protein, we made use of multi-state structure calculation in CYANA [21, 22], which has recently been extended to PREs [58]. This is a modified structure calculation protocol which creates multiple copies of the system in question (states) and allows distance restraints to be satisfied by averaging over these states. These are known as ambiguous restraints. Importantly, the distances are averaged as $\langle 1/r^6 \rangle$ which implies that states with short distances dominate the effective distance. Thus, for example a single state with a sufficiently short distance can satisfy the distance restraint derived from a strong PRE even in the presence of other states which do not satisfy that distance limit. Namely, the effective averaged distance $D^\ast _{ab}$ from a nuclear spin $a$ to the electron spin of spin label $b$ that is used in a PRE distance restraint is computed as:


(6) \begin{eqnarray*}
D^\ast _{ab} = \left( \sum _{i-1}^N \left( d^i_{ab}\right)^{-6}\right)^{-1/6}
\end{eqnarray*}

where $d^i_{ab}$ is the distance in state $i$, and $N$ is the number of states. This is the same averaging expected for distances derived from NOEs measured with a dynamically averaging ensemble. Note that this method makes a number of simplifications to the expected mathematical dependence: The spin label position is represented at the average position derived by an unrestrained ensemble of spin label conformations at the label position (vide infra), the number of states of the protein used is much lower than the expected number of conformations averaged during the PRE measurement in solution, and all states are assumed to have equal population.

##### Incorporation of the spin label and calculation of the starting structure for multi-state CYANA

The spin label was incorporated in the CYANA calculation by an approach built upon a previous study [59]. The structures of the RRM domains of interest were recalculated using restraints derived from NOEs defined in the structures of the individual RRMs (PDB numbers: 1X4A and 2O3D), while replacing the native cysteines and mutated cysteine residues with the new residue CYSM, which contains additional atoms for the MTSL attached to the cysteine. The CYSM spin label atoms were unconstrained and sampled the available conformational space in the generated ensembles of 150 conformers. We considered atom N1 a proxy for the unpaired electron, since the unpaired electron is distributed between the N and O. We then computed the average distance of the spin-label atom N1 to the CA atoms of seven residues (CA) within the folded regions of the domain the spin label was attached to. These distances were restrained to the determined average distance $\pm 0.2$ Å in the following structure calculations. In the next step, we introduced a dummy atom, Q8, to represent the centre of the spin cloud: we replaced the CYSM residue by the native residue with a short chain of non-space-filling dummy atoms (Q1–Q8) attached to the residue’s CA atom. This was accomplished by incorporating additional residues (CYSL, TYRL, SERL, and THRL) into the CYANA library that include these dummy atoms and changing the sequence file accordingly. We then recalculated the individual RRM structures including the atoms Q1–Q8 for each spin label and the 7 tightly defined upper and lower distance restraints from Q8 to the seven CA atoms in the RRM mentioned above to position each dummy atom Q8 at the centre of the spin cloud. The final structure of the domain including these eight dummy atoms for each spin label served as a rigid model of the RRM and the angles of these residues including Q1–Q8 were fixed to the determined values for subsequent calculations of the full-length system, which included residues 1–196 of SRSF1, the Q1–Q8 atoms for each spin-labelled residue, and upper and lower distance limits derived from PRE restraints from each Q8 atom to each amide for which PRE restraints were determined. Similar to the approach described in [59] this method accounts for the average position of the unpaired electron of the spin label but does not consider dynamics of the spin label. The new approach using Q1–Q8 to position the centre of the spin clouds dramatically reduces the number of coordinates required (only 8 additional coordinates per spin label which are fixed during calculations) compared to the previous method which for each spin label required long flexible linkers of approximately 50 residues containing dummy atoms followed by a GLY residue whose CA atom was constrained to place it at the centre of each spin cloud [59]. This approach was critical for the convergence of multi-state calculations which included up to 10 copies of the protein as described below. To obtain the starting structure for each multi-state CYANA calculation with all the spin cloud atoms Q8 correctly positioned, 300 multi-state structures were generated by duplicating all the 7 CA–Q8 upper and lower distance restraints for each spin label, plus the NOE upl restraints obtained for the individual RRM domains which were defined for all copies (states) of the protein and the 20 with the lowest target function values were evaluated for structural statistics. The structure with the best target function and the lowest number of violations was then used as the starting structure for the CYANA multi-state calculation.

##### Multi-state CYANA calculation

We adapted the demo/enoe/PREP.cya macro from CYANA 3.98.15 to prepare all the input data for the CYANA multi-state calculation [21, 22]. The data were prepared according to the number of states that were to be calculated. The raw ensemble generation was performed on the ETH Euler compute cluster by adapting the demo/enoe/2_CALC_multi-state.cya macro from CYANA 3.98.15. The starting structure (described in the previous section) was read, and the structures of the individual RRMs were fixed (residues 16–88 for RRM1 and residues 120–196 for RRM2). This also fixed the angles of the Q1–Q8 dummy atoms which placed each spincloud. The linker segment (89–119) however was flexible.

To determine the number of states required to satisfy the full set of PRE restraints, tests were performed, calculating from 1 to 10 states (2000 structures with 200 000 annealing steps, best 20 structures selected). The number of states that exhibited the best structural statistics without elongated structures was selected for the final ensemble calculation. Elongated structures showed extended linkers and generally did not help to satisfy the PRE restraints. These appeared when the number of states was too high, suggesting that the additional states included in these calculations were not needed in order to satisfy the PRE restraints. The final calculation was performed for 5000 structures with 200 000 annealing steps, and the best 20 structures were selected. This resulted in 20 structures for each of the 7 states. We then used an adapted demo/enoe/SPLIT.cya macro to remove the linkers between the individual states, and to obtain the final CYANA model with the correct residue numbering.

### Ensemble comparison

Different ensembles for the same state as well as the ensembles of free SRSF1$_{\Delta \mathrm{RS}}$ and of the complexes are compared by the similarity measure $s$ introduced in [60], which ranges between 0 when two single-conformer ensembles with distinct conformers are compared and 1 when two identical ensembles are compared. Ensembles are compared visually by superimposing all conformers onto RRM2 (121–195) and colour-coding (RRM1 in red, RRM2 in blue). Conformer weight is encoded by transparency, with the conformer with maximum weight $w_\mathrm{max}$ being represented fully opaque ($\alpha = 1$) and opaqueness of other conformers given by $\alpha = w_c/w_\mathrm{max}$. Quantitative ensemble comparison and automated generation of visualization scripts were performed in MMMx [60] and visualization was performed in MMM [26].

### Disorder parameter

The distance root-mean-square deviation $D_{kl}$ between two conformers with indices $k$ and $l$ is a measure for their difference in global shape. $D_{kl}$ has the properties of an Euclidean distance. For an ensemble with $C$ conformers, the $C(C-1)/2$ values of $D_{kl}$ define a point set in an Euclidean space, which can be computed by multi-dimensional scaling. We call this space abstract conformer space (ACS) [57]. The point set has a radius of gyration $R_\mathrm{g,ACS}$ in ACS, which has the same unit as a real-space distance, since the $D_{kl}$ also share this unit. $R_\mathrm{g,ACS}$ is a measure for global shape diversity in the ensemble of conformers and hence for disorder. As the $D_{kl}$ for conformers in a random-coil ensemble increase with increasing real-space radius of gyration $R_\mathrm{g}$ of the peptide chain, it is convenient to scale the disorder measure by $R_\mathrm{g}$, We thus arrive at the disorder parameter $\delta = R_\mathrm{g,ACS}/R_\mathrm{g}$. Real-space radius of gyration for ensembles was computed by considering only C$^\alpha$ atom positions and averaging as $R_\mathrm{g} = \sqrt{\sum _{c=1}^C w_c R_{\mathrm{g},c}^2}$, where $C$ is the number of conformers in the ensemble, the $w_c$ are their weights, and the $R_{\mathrm{g},c}$ are the radii of gyration of the individual conformers. In recent work [57], we established that disorder parameters range between about 0.1 in NMR ensembles of folded proteins, such as ubiquitin, and 0.6–0.8 in ensembles of fully disordered proteins where the chain assumes random-coil character.

## Results

### Tandem SRSF1$_{\Delta \mathrm{RS}}$ RRMs are neither independent from each other nor strongly interacting

The structures of isolated RRM1 and RRM2 in free form (PDB 1X4A, 2M7S/2O3D) and in complex with RNA (PDB 6HPJ, 2M8D) have been solved [14, 39, 55]. RRM1 binds preferentially to CN motifs (N stands for A, C, G, or U nucleotide), with the highest chemical shift changes observed for CA, using its canonical $\beta$-sheet interface, whereas the pseudo-RRM2 binds GGA motifs using a conserved heptapeptide in the $\alpha$1-helix. To achieve sufficient solubility and keep the free protein dispersed, we used a truncated construct lacking the RS domain (SRSF1$_{\Delta \mathrm{RS}}$, residues 1–196) and containing a GB1 tag and two point mutations Y37S and Y72S (Fig. 1). These point mutations have previously been shown not to affect RNA binding [14].

First, the folding of this construct was compared to that of the isolated RRMs. Using $^{1}$H-$^{15}$N heteronuclear single quantum spectroscopy (HSQC) NMR spectra, we observed that the RRMs adopt the same fold when they are linked in tandem (Supplementary Fig. S2A), and as expected some chemical shift differences were observed for the residues adjacent to the interdomain linker (Supplementary Fig. S2B). To assess the flexibility of the linker, we conducted $^{15}$N-spin relaxation experiments, which are sensitive to the backbone dynamics [61, 62]. Low $\lbrace ^{1}\mathrm{H}\rbrace ^{15}\mathrm{N}$ NOE values, indicating subnanosecond dynamics and high flexibility, were observed for residues located near the N-terminus of the protein (1–15) and the inter-RRM linker, as evidence of flexible protein regions (Supplementary Fig. S2C). In contrast, $\lbrace ^{1}\mathrm{H}\rbrace ^{15}\mathrm{N}$ NOE values for the folded regions of both RRMs were higher (around 0.7–0.8) as expected for the more rigid folded domains. The $\lbrace ^{1}\mathrm{H}\rbrace ^{15}\mathrm{N}$ NOE values exhibit some correlation to the AlphaFold3 predicted local distance difference test (pLDDT), although this correlation is not linear (Supplementary Fig. S2D).

In addition, sets of longitudinal $T_1$ and transverse $T_2$ relaxation times were measured and used to calculate the rotational correlation times $\tau _\mathrm{c}$, which provide information on the global motion (Supplementary Fig. S2E) [62]. In the single RRM constructs, the RRMs have $\tau _\mathrm{c}$ values of $7.5 \pm 0.5$ ns and $6.8 \pm 0.7$ ns, calculated for residues 16–88 of RRM1 and 120–195 of RRM2, respectively. Essentially the same values were obtained analysing the individual RRMs mixed in the same sample ($7.2\pm 1.2$ ns for RRM1 and $7.0 \pm 0.8$ ns for RRM2), suggesting that under these conditions the two RRMs still behave as independent domains. For folded domains, the correlation time is, to a good approximation, proportional to their molecular volume [63]. Thus, a doubling of $\tau _\mathrm{c}$ would be expected for strongly interacting domains, as observed for example for the two RRMs of UP1 [45] or for the two C-terminal domains of PTB [64].

Compared to the single RRMs, for SRSF1$_{\Delta \mathrm{RS}}$, we observe an increase of the $\tau _\mathrm{c}$ value to $11.0 \pm 1.9$ ns for RRM1 and $9.6 \pm 1.5$ ns for RRM2. While the increase indicates that the tandem domains do not tumble completely independently from each other, the $\tau _\mathrm{c}$ values differ and are lower than the sum of the values for the individual RRMs ($\approx 14$ ns). Previous work has shown a drag effect of interdomain linkers of $\approx$25 residues can cause an increase of $\tau _\mathrm{c}$ of the linked domains by up to $\approx 30$% [65], which is on the order of what we observe. Therefore, we hypothesized that the linked domains do not interact strongly, and the tandem cannot be described by a single conformation. This result is in line with the AlphaFold3 predicted aligned error (PAE) matrices for full-length SRSF1 and for our construct (Supplementary Fig. S3). The PAE values for pairs of residues in the two different RRMs are lower than the ones between the linker and either RRM but much higher than the ones for pairs of residues within the same RRM. Likewise, the PAE matrix for our construct (Supplementary Fig. S3B) may indicate some interaction between the GB1 solubility tag and RRM1, but such interaction is predicted not to be strong.

Correlation times were also measured for the protein–RNA complexes (Supplementary Fig. S2F), and a slight increase in $\tau _\mathrm{c}$ values was observed for both RRMs ($\approx 11.5$ ns for each domain). As for the free protein, this result indicates that, when the protein is bound to the different RNAs, the two RRMs are not in a fixed configuration but adopt multiple dynamically interchanging conformations.

We also analysed NMR chemical shifts of the HN, N, C$\alpha$, and C$\beta$ atoms in terms of secondary structure propensities [66, 67]. As illustrated in Supplementary Fig. S4 for free SRSF1$_{\Delta RS}$, the inter-RRM linker (residues 91–120) is coil-like throughout with negligible propensities of $\alpha$-helical or $\beta$-strand secondary structure. This agrees with an earlier finding that the linker is dynamic [15].

### DEER shows that SRSF1$_{\Delta \mathrm{RS}}$ adopts different conformations in the free and RNA-bound states

#### DEER distance distributions are generally broad

To quantify the extent of disorder and conformation changes upon binding, we probed SRSF1$_{\Delta \mathrm{RS}}$ by DEER measurements. This technique reports on the distance distribution between two paramagnetic centers in the range of 15 Å to $\approx 100$ Å [30]. We introduced paramagnetic centers by site-specific attachment of a methanethiosulfonate spin label (MTSL) to native or engineered cysteines. We selected three reference sites in each RRM; C16, Y37, and Y72 in RRM1 and S126, C148, and T169 in RRM2. These sites were chosen considering their accessibility for the spin label attachment and minimizing the potential for disrupting the structure or the RNA binding. We selected seven double cysteine mutants combining the reference sites and characterized each with DEER experiments in three distinct states: SRSF1$_{\Delta \mathrm{RS}}$ in the free form, in complex with RNA9, and in complex with RNA12 (Supplementary Fig. S5). Note that during all the experiments, two cysteines were present at a time, requiring in some cases to mutate the native cysteines to the non-reactive amino acid alanine, e.g. C16 S126C is combined with C148A. Henceforth, we will only explicitly indicate the sites directly involved in the distance measurements, e.g. C16 S126C. 

**Figure 2.**
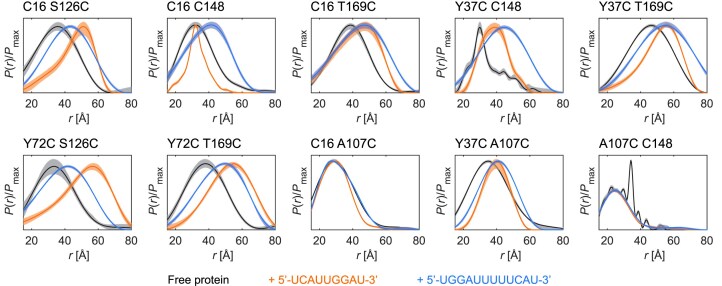
Intra-protein distance distributions. Inter-RRM (C16 S126C, C16 C148, C16 T169C, Y37C C148, Y37C T169C, Y72C S126C, and Y72C T169C) and RRM-linker (C16 A107C, Y37C A107C, and A107C C148) distance distributions were measured for SRSF1$_{\Delta \mathrm{RS}}$ in the free state (black), in complex with RNA9 (orange), and in complex with RNA12 (light blue). The spin-label positions are indicated above each panel. Solid lines are the medians and shaded areas the 95% confidence intervals [50]. The distance distribution of free A107C C148 was normalized with respect to the broad feature to facilitate the comparison. Primary DEER data are reported in Supplementary Fig. S5A.

All inter-domain distance distributions show pairwise differences between the free SRSF1 and the two complexes (Fig. 2). Except for C16 C148, where free SRSF1 and the complex with RNA9 have a similar mean distance but different width, the mean distances always increase upon RNA binding. For C16 T169C and Y37C T169C, both complexes have a similar mean distance, while for the other mutants, either the distance in the complex with RNA9 (C16 S126C, Y72C S126C, and Y72C T169C) or in the complex with RNA12 (C16 C148 and Y37C C148) is more extended. Together with the NMR data, these experiments reveal that the protein adopts a different set of conformations when RRM1 binds upstream or downstream of the RRM2-binding site. The distance distributions are generally broad, highlighting the conformational freedom provided by the linker. There are two exceptions: the distribution C16 C148 for SRSF1 in complex with RNA9 is relatively narrow and the distribution of Y37C C148 in the free form presents a bimodality with a broad feature overlaid by a narrow feature.

To probe the positioning of the linker with respect to the RRMs, we measured for each system three DEER traces connecting A107C in the linker to either C16, or Y37C in RRM1, or C148 in RRM2 (Fig. 1). The Y37C A107C distribution shows clear differences between the free and bound forms. The other linker-RRM distance distributions display only minor differences upon RNA binding. The distribution for free A107C C148 contains a narrow feature, suggesting the presence of an interaction between RRM2 and the linker for a small fraction of proteins. This feature corresponds to $\approx 11$% of the area under the distribution. For free full-length SRSF1 (measurements to be reported elsewhere), we did not observe this narrow feature. This difference could be traced back to using a buffer with pH 7.4 for full-length SRSF1 instead of the buffer with pH 6 used for SRSF1$_{\Delta \mathrm{RS}}$. When performing the measurements with spin-labelled SRSF1$_{\Delta \mathrm{RS}}$ from the same batch at pH 6.0 and 7.4 under otherwise identical conditions, we observe the change in distance distribution as well (Supplementary Fig. S6). This pH dependence might suggest interaction with a protonated histidine at low pH, as p*K*a values of the imidazole sidechain range between 5.5 and 7 depending on chemical context. If so, H140 in RRM2 would be the only candidate. However, in the absence of other evidence we can only speculate about such an interaction. As pH in cell is closer to 7.4 than to 6.0, we refrained from further study of this feature.

Lastly, we also performed intra-RNA and protein–RNA DEER measurements by introducing spin labels at the 5′- and 3′-termini (see Supplementary Fig. S5B–D for primary DEER data). Unsurprisingly, intra-RNA measurements performed on the spin-labelled RNAs in complex with unlabelled SRSF1$_{\Delta \mathrm{RS}}$ reveal that RNA12 is more extended and flexible than RNA9 (Supplementary Fig. S7A and C). In addition, we measured distance distributions from the RNA termini to C16 in RRM1, A107C in the linker, or C148 in RRM2, yielding a total of six additional measurements per protein–RNA complex (Supplementary Fig. S7B and D). The distance distributions of RRM1 and RRM2 to the RNA ends closest to their binding motifs are narrow, confirming the expected RNA binding orientation. The distributions for the distances from the RRMs to the RNA-ends further away are broad, in line with the broadness seen in the intra-RNA distance distributions. The distance distributions involving the linker are all broad and do not hint at any linker-RNA interactions.

#### Inter-RRM PRE differs between the labelling sites

Concurrently to the DEER experiments, PRE NMR experiments were conducted to obtain information on distances ranging from approximately 12 to 25 Å [33, 68]. The PRE technique probes the effect of a paramagnetic centre on relaxation of protons in the vicinity, with high PRE effects corresponding to short distances and low PRE effects corresponding to longer distances. We attached MTSL to the cysteines C16, Y37C, and Y72C in RRM1 and S126C, C148, and T169C in RRM2. Each construct contains only one label in RRM1 or one label in RRM2. These experiments are complementary to recent PRE experiments on two RNA complexes of a similar SRSF1 construct with a single label site E120C at the C terminus of the linker where it attaches to RRM2 [15]. To characterize interaction of the RRMs in more detail and provide localization information for the full linker, we use three label sites in each RRM. We extend the PRE measurements to free SRSF1$_{\Delta \mathrm{RS}}$ in order to study interaction between the tandem RRMs in the absence of RNA.

**Figure 3.**
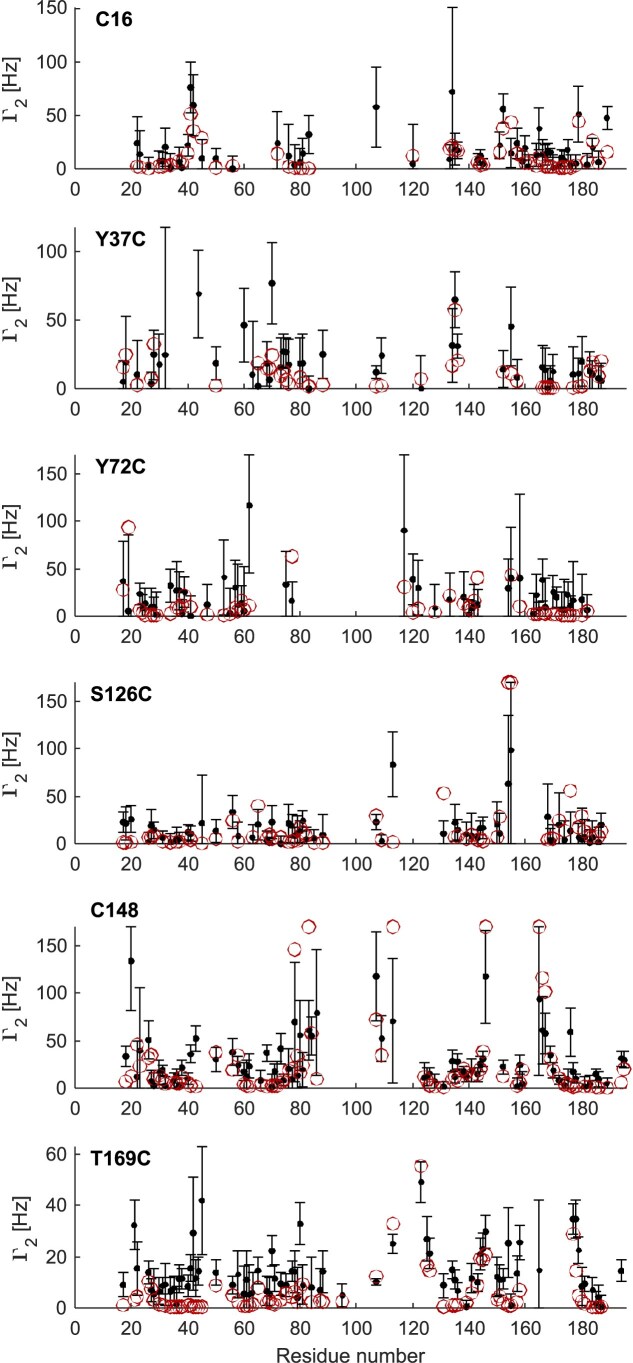
NMR PRE data for free SRSF1$_{\Delta \mathrm{RS}}$. PRE rates are shown as black points with error bars. Backcalculated data (red open circles) are weighted means for the RigiFlex superensemble. Backcalculated data are not shown for residues where it was capped at 170 s$^{-1}.$.

The transverse relaxation enhancement (PRE rate) was analysed for each residue: the values are in the range from 0 to 170 Hz, with the lower limit corresponding to an undetectable PRE effect and the upper limit corresponding to signal broadening beyond detection at approximate mean distances of 12 Å or shorter. Analysis of the PRE results for the free protein reveals PRE effects both within the domain where the spin label was attached and the other RRM (Fig. 3). As expected, a stronger intra-RRM PRE effect was observed for residues close to the spin label. PRE effects were also observed in the linker, indicating a proximity to one of the two RRMs. When analysing the RNA-bound states (Supplementary Fig. S8), we observed that the PRE effects were similar to those observed for the free protein. Although the PRE rates change, these changes are not systematic and mostly do not exceed uncertainty. Together with the significant differences of inter-RRM PRE between labelling sites, this indicates that those conformers where the two RRMs are closest to each other are similar in the free and bound states.

## Integrated ensemble modelling of RNA binding

### Complementarity of PRE and DEER restraints

We aim to develop a robust and validated workflow for characterization of RNA binding at the level of molecular structure. This requires inference of ensemble models because promiscuous RNA binding by multi-functional proteins relies on protein flexibility. When such flexibility is substantial, most established approaches in structural biology either fail or provide only partial information. This implies the need for integrated structural biology [69] where restraint sets from different techniques are combined.

Such an integrated modelling approach requires the selection of appropriate complementary methods depending on the problem class. This is necessary, first, because not all techniques are applicable in all environments and, second, because adding additional restraints is economic only if they provide substantial additional information. In the context of RNA binding, we are dealing with proteins that feature both well-defined IFRs, such as RRMs, and IDRs, such as inter-RRM linkers or terminal low-complexity domains. In the current study we removed the low-complexity RS domain, whereas we still aim to develop a modelling approach that can deal with long terminal IDRs. In recent work on heterogeneous nuclear ribonucleoprotein A1 (hnRNP A1), we demonstrated that the combination of DEER distance distribution restraints, PRE restraints, and small-angle X-ray scattering (SAXS) restraints is a suitable approach for long terminal IDRs [70]; whereas in recent work on the complex of polypyrimidine tract-binding protein 1 with an internal ribosome entry site of encephalomyocarditis virus (PTBP1/EMCV-IRES), we demonstrated that the combination of DEER distance distribution restraints with SAXS and small-angle neutron scattering (SANS) is a suitable one for distributed arrangement of RRMs [59]. In both cases, we relied on a classical structural biology technique, namely high-resolution NMR, for determining the structure of the IFRs, which were then treated as rigid bodies in ensemble modelling. This rigid-body approach is justified if NMR chemical shifts of the individual RRMs and of the RRMs in the modelled constructs agree, as they do in the case at hand. If substantial chemical shift changes are observed, integrity of the RRM structures needs to be checked by other means such as by measuring DEER restraints for pairs of spin labels at sites in the same RRM.

For both hnRNP A1 and PTBP1/EMCV-IRES, similar ensembles were obtained when using only DEER restraints or all restraints. Further, sufficiency and internal consistency of DEER restraint sets could be validated [38]. However, pure DEER restraint sets have two limitations. First, DEER is not sensitive to distances shorter than about 15–20 Å, so that short-range interactions enter the ensemble only indirectly by combination of molecular geometry restraints of proteins with long-range information. Second, DEER restraints can be obtained only in frozen solution. This is more problematic than in determination of IFR structure by X-ray crystallography at low temperature or cryo-electron microscopy, since the conformational ensembles are governed by weaker interactions than is the tertiary structure of IFRs. Whereas cold denaturation of RRMs is not expected, partial unfolding of RRMs might occur, for instance, in condensed phases formed by liquid–liquid phase separation. For these reasons, it is preferable to complement DEER restraints by at least one technique that is applicable at ambient temperature in liquid solution. This can be SAXS, SANS, PRE, or single-molecule fluorescence energy transfer (smFRET) [71]. Among these techniques, PRE is distinguished by also providing short-range information and NMR chemical shift information that can prove structural integrity of the RRMs. PRE is also the technique that is most easily applicable in the condensed phases that arise from liquid-liquid phase separation of RNA-binding proteins [72]. Therefore, we develop a minimal integrated approach based on DEER and PRE restraints, without precluding the use of SAXS, SANS, or smFRET in cases where this is feasible and complementary information is expected. NMR chemical shifts for determining secondary structure propensities [66, 67] are straightforward to obtain when NMR is used to acquire PRE restraint sets. If such propensities are significant, they can be incorporated in the RigiFlex branch of our approach by biasing Ramachandran angle sampling in Flex [73].

While an approach based only on PRE restraints is easier to realize and certainly useful, it also has limitations. PRE is sensitive to distances in the range 12–25 Å, in favourable cases up to 35 Å with lanthanide instead of nitroxide labels. Given the size of RRMs, the length of RRM linkers, and the dimension of terminal IDRs, one cannot expect to obtain an ensemble that is well-restrained on all relevant length scales when using only PREs. DEER distance distribution restraints, SAXS, SANS, and smFRET data can provide complementary restraints. The label-free small-angle scattering techniques provide general shape information, while DEER and smFRET can provide site-pair resolved information and thus a larger number of better resolved restraints. Among these techniques, DEER provides better resolved information on distance distribution shape and width and can be more easily interpreted in terms of absolute distance. Here, we aim for ensemble models that characterize flexible binding in a static picture. For a minimal approach, DEER appears better suited than smFRET, whereas complementation by smFRET is required for understanding the dynamic aspects of binding [71].

### Size of the restraint set

In classical structural biology, quality of a model can be estimated from the number and resolution of restraints. In general, the restraint sets are much larger than in ensemble structural biology, because high resolution of well-defined structure implies that many signals can be resolved. At first sight, solving the more complex problem of ensemble structure with less restraints appears hopeless. However, as high structural resolution implies that more restraints can be gathered, the low structural resolution of flexible systems implies that less restraints are required for characterization.

The required number of restraints thus depends on flexibility, which is heterogeneous across the structure and unknown before the ensemble is modelled. Hence, only with an ensemble already in hand, can we determine whether this ensemble is sufficiently restrained. We do so by leaving out individual restraints and by observing how this changes the ensemble and how well the left-out restraint is determined by an ensemble computed with only the other restraints (jackknife resampling) [38]. Systematic jackknife resampling provides an ensemble of ensembles from which uncertainties of any observable can be estimated.

### Size of the conformer pool and ensemble

Even when discretizing the problem of IDR conformation by assuming that each Ramachandran angle pair can assume one of only three canonical states, the number of possible conformers of RNA-binding proteins is much larger than a feasible pool size. For the 30-residue inter-RRM linker, this already reduced sampling scheme implies >10$^{13}$ possible conformers. Neither can we hope to gather sufficient experimental information for specifying >10$^{13}$ weights, nor do we need such a large ensemble for predicting the values of all potential observables. The problem is similar to the one of statistical mechanics, where on accepts that the full information on the system is not accessible and aims to predict the information that can be experimentally obtained. An ensemble is representative if it predicts all restraints within their experimental uncertainty. This limits the required ensemble size. Ensemble reweighing in MMMx implements this idea by discarding a conformer from a pool if its weight is so small that this conformer does not influence the values of observables beyond their uncertainty.

This approach does not yet answer the question on the required pool size. Assuming that the sampling is random and that a huge number of samples (in our case on the order of 10$^{13}$) would access the whole conformational space, the problem can be solved by testing for convergence. To this end, we stepwise add blocks of approximately 100 conformers to the preliminary ensemble, repeat reweighing and observe the change in fit quality. Pool size is sufficient if the figures of merit for all restraint sets (in our case DEER and PRE) have converged on the order of their remaining fluctuation.

### The two RRMs interact in free SRSF1$_{\Delta \mathrm{RS}}$

#### Ensemble models for free SRSF1$_{\Delta \mathrm{RS}}$

To assess the quality of the initial conformer pools [34] and the information content of the restraint sets, we modelled ten ensembles of free SRSF1$_{\Delta \mathrm{RS}}$ by integrating EPR DEER and NMR PRE restraints (Table 1). To this end, we generated pools of 2000 unrestrained conformers by the torsion-angle dynamics approach in CYANA [35], by AF3 runs with different random seeds [37], and by the Rigi module of MMMx [36]. The initial Rigi pool specifies only relative arrangement of the two RRMs based on the EPR distance distributions (Supplementary Fig. S1). For 764 of these rigid-body arrangements, insertion of the linker residues 91–120 by the Flex module [73] failed, so that we ended up with a RigiFlex pool of 1236 conformers. Sampling in the AF3 pool is potentially enhanced by information on spatial relation between the RRMs from the pairformer module as indicated by the PAE matrix, which predicts the expected position error of residue $x$ when structures are superimposed at the N, C$\alpha$, and C atoms of residue $y$ (Supplementary Fig. S3). We have recently demonstrated that the PAE matrix is related to the ensemble aligned uncertainty matrix, and thus also predicts the distribution width of the C$\alpha$ atom of residue $x$ upon backbone superposition at residue $y$ [74]. Sampling in the RigiFlex pool is enhanced by taking into account distance distribution restraints in both the Rigi and the Flex step.

**Figure 4.**
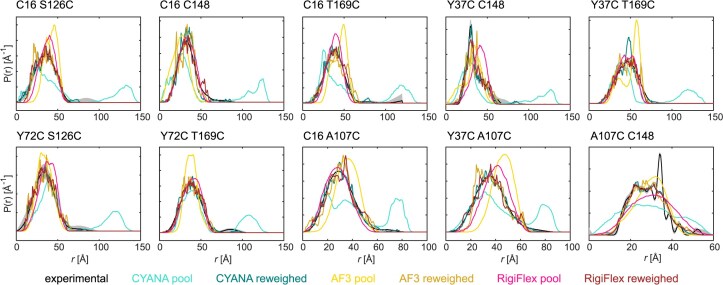
Fit of distance distributions for free SRSF1$_{\Delta \mathrm{RS}}$. Shown are the experimental distributions (black lines) with 95% confidence intervals (grey area) and backcalculated distributions from the initial CYANA pool (light cyan), AF3 pool (yellow), and RigiFlex pool (magenta). Backcalulated distributions for the CYANA multi-state ensemble (dark cyan) or after ensemble reweighing are displayed in darker shades (AF3 goldenrod, RigiFlex chocolate).

Indeed, the EMD as a measure of DEER fit quality is lowest in the initial RigiFlex pool (3.3 Å), despite its smaller size, and largest in the unrestrained CYANA pool (16.9 Å). The reduced inter-RRM PAE in the AF3 predictions corresponds to a decrease of the EMD (5.8 Å) with respect to the unrestrained CYANA pool. Comparison of the backcalculated and experimental distance distributions (Fig. 4) reveals that the larger EMD of the AF3 and, especially, the CYANA initial pool stems from conformers with large inter-RRM distances that violate the experimental restraints. Note that for the RigiFlex pool the EMD is not much larger than the combined experimental and modelling uncertainties, as the error contribution from rotamer library modelling of the label conformations amounts to $2\ldots 3$ Å [75].

The pool sizes are sufficient as indicated by convergence of the EMD for the distance distributions and $\chi ^2$ for the PRE when adding new blocks of conformers to the pool, as exemplified for the RigiFlex ensemble in Supplementary Fig. S9. After reweighing the initial ensembles against both the DEER and PRE restraints, all three approaches led to similar fit quality of the backcalculated data (Table 1). For the CYANA and RigiFlex ensembles, the EMD of 0.7 and 0.8 Å, respectively, is within the expected uncertainty. The AF3 approach leads to a slightly larger EMD of 1.3 Å. Given the experimental uncertainty of the distance distributions (grey bands in Fig. 4) that adds to label modelling uncertainty, the differences in EMD are too small for preferring any approach. We find the best PRE fit with RigiFlex ($\chi ^2 = 5.699$), followed by CYANA (6.348), and AF3 (6.544). Again, the differences are hardly significant, given that a balanced fit corresponds to a $\chi ^2$ value of 1. The relatively large $\chi ^2_\mathrm{PRE}$ may be partially due to uncertainty in modelling the spin label rotamer distribution. However, we note that the model for backcalculation assumes that interconversion between conformers is slow on the NMR time scale, while the time scales of large-scale conformational change and relaxation delays in PRE experiments may overlap [51]. Limitations of this approximation may also add to the deviation between experimental and backcalculated values.

For the unrestrained CYANA pool, we find that fitting exclusively the DEER restraints only very slightly improves agreement of the backcalculated PRE with experiment. In contrast, fitting only the PRE restraints improves agreement of backcalculated DEER restraints moderately from an EMD of 16.9 to 10.6 Å indicating complementarity of the two restraint sets. For the RigiFlex pool, which already contains information from DEER, fitting to only PRE restraints worsens the agreement with DEER restraints from an EMD of 3.3 to an EMD of 5.1 Å.

**Figure 5.**
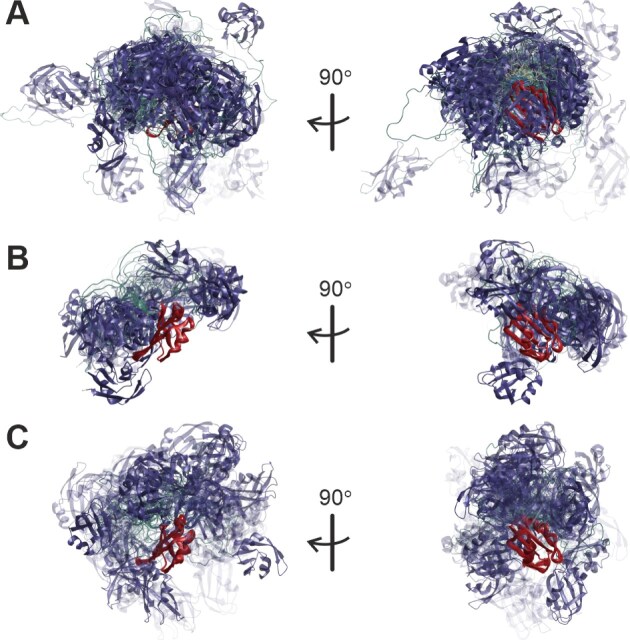
Ribbon models of reweighed ensembles for free SRSF1$_{\Delta \mathrm{RS}}$. Conformers are superimposed on RRM1 (red) with transparency encoding weight. The linker is shown in cyan and RRM2 in blue. All ensembles were fitted integratively to EPR DEER and NMR PRE restraints. (**A**) Ensemble obtained from the CYANA pool. (**B**) Ensemble obtained from the AF3 pool. (**C**) Ensemble obtained from the RigiFlex pool.

The three reweighed ensembles share some common features, while also exhibiting differences (Fig. 5). The ensemble derived from the RigiFlex pool (panel C) is least compact ($R_\mathrm{g} = 21.1$ Å Table 1), whereas the one derived from the AF3 pool (panel B) is most compact ($R_\mathrm{g} = 19.7$ Å). The latter finding might have been expected, as AF3 is trained on crystal structures and thus biased towards the Anfinsen limit of a single populated conformer. The RigiFlex ensemble (panel C) is derived from the pool that best samples the relevant part of conformation space, as follows from the substantially better agreement of this pool with both the distance distributions and the PRE data as compared to the other two initial pools. Hence, one might prefer this ensemble. However, when we consider the agreement of only the reweighed ensembles with experiment, we find no reason for discriminating against the ensemble derived from the CYANA pool.

Since AF3 was reported to occasionally predict wrong chirality [37], we generated Ramachandran plots for the inter-domain linker region (residues 91–120) for the AF3 ensemble and, for comparison, for the RigiFlex ensemble. These plots, shown in Supplementary Fig. S10, reveal some differences in coil representation of the linkers between the two ensembles. They do not indicate substantial population of Ramachandran angles far from favoured regions for either of the two approaches.

Finally, we tested for the influence of the GB1 solubility tag on the ensemble by generating a second AF3 pool of 2000 conformers for the construct without this tag that we had also not considered in generating pools with approaches other than AF3. The reweighed AF3 ensemble without the GB1 tag has a similarity of $s = 0.9875$ to the one with the GB1 tag. Comparison to Table 2 reveals that differences in the ensembles obtained by different modelling approaches are larger than the influence of the GB1 tag.

**Table 2.** Similarities of ensemble models for free SRSF1$_{\Delta \mathrm{RS}}$ obtained from various initial conformer pools and by the CYANA multi-state (MS) method

Pool	CYANA	AF3	RigiFlex	CYANA MS
CYANA	1	0.9175	0.9633	0.9217
AF3	0.9175	1	0.9045	0.9036
RigiFlex	0.9633	0.9045	1	0.8905
CYANA MS	0.9217	0.9036	0.8905	1

All three reweighed ensembles are more compact than the unrestrained CYANA pool, as is apparent from Fig. 4 and from Supplementary Fig. S11. They all have mutual similarities $s$ higher than 0.9 (Table 2), indicating no major inconsistencies. However, the AF3 ensemble differs more strongly from the two other ensembles than is observed for the majority of ensemble pairs for the same protein in the same binding state in the Protein Ensemble Database [60].

#### The restraint set for free SRSF1$_{\Delta \mathrm{RS}}$ is consistent and sufficient

All three reweighed ensembles fit the experimental data to a reasonable extent, yet they differ significantly from each other. This raises the question of consistency and sufficiency of the restraint set. We observe a low loss of merit $L < 0.1$ upon balancing the EPR DEER and NMR PRE restraints for all three initial pools. Likewise, a fit of the unrestrained CYANA pool to only the EPR distance distributions or only the PRE restraints is only slightly better than the simultaneous fit from the RigiFlex pool. This suggests that the two restraint sets are largely consistent with each other, despite the fact that the PRE restraints were acquired in liquid solution at ambient temperature and the DEER measurements were done in glassy frozen solution at 50 K. The internal consistency of the distance distributions with each other is also indicated by the small EMD and an overlap deficiency that is on par with our previous work on hnRNP A1 [38] and better than in our previous work on the PTBP1/EMCV-IRES protein–RNA complex [59].

**Figure 6.**
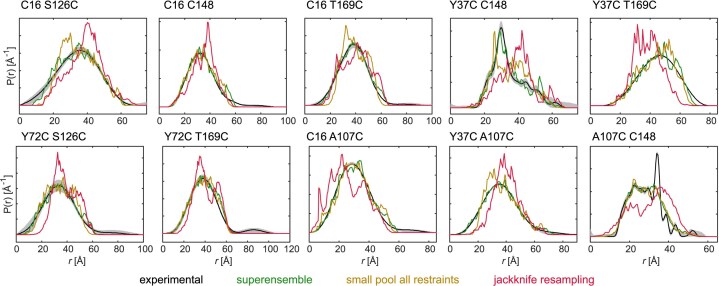
Distance distribution fits in jackknife resampling for SRSF1$_{\Delta \mathrm{RS}}$. Shown are the experimental distributions (black lines) with 95% confidence intervals (grey area) and backcalculated distributions from the reweighed superensemble (green), the ensemble obtained by reweighing against all restraints from a RigiFlex pool with 292 conformers (goldenrod), and by reweighing a RigiFlex pool computed without this restraint and omitting the restraint also in reweighing (magenta).

To test for sufficiency of the set of distance distributions, we performed jackknife resampling. To limit computational expense, we used smaller initial pools of only 500 rigid-body arrangements and repeated the fit with all restraints for this pool size (golden lines in Fig. 6). As expected, the backcalculated distance distributions for the restraints that were not used in reweighing (magenta lines) fit the experimental distributions somewhat worse. Most of them are still in rather good agreement with the experimental data, with the exception of double mutant Y37C C148, where the modal distance shifts considerably and Y37C T169C, where the EMD increases to 7 Å (Supplementary Table S1). Altogether the outcome is somewhat worse than for hnRNP A1 [38]. However, all validation ensembles have a similarity of at least 0.9785 to the reweighed superensemble (vide infra, Supplementary Table S1), which is higher than the similarity between the reweighed RigiFlex and CYANA ensemble (Table 2). This suggests that the restraint set is sufficient to specify the ensemble structure. We tentatively assign the differences between the CYANA and RigiFlex ensemble to different sampling of conformation space in the initial pools.

Sampling is enhanced when pooling the RigiFlex ensemble with all validation ensembles to a superensemble. The total size of the underlying pool of conformers thus increases from 1236 to 4309 conformers. Indeed, reweighing of this superensemble provides an even better fit to the experimental distance distributions (green lines in Fig. 6), whereas the fit of the PRE restraints improves only insignificantly (Table 1). We consider this reweighed superensemble as the best estimate for the solution structure of free SRSF1$_{\Delta \mathrm{RS}}$ that we can currently obtain. Similarity between this ensemble and the original RigiFlex ensemble is very high at 0.9967.

The superensemble is an ensemble of ensembles. We have computed the radius of gyration $R_\mathrm{g}$ and the disorder parameter $\delta$ for each of the underlying validation ensembles to measure uncertainty of these quantities (Table 1). In particular the disorder parameter is very well defined with a 95% confidence interval of only 0.020, whereas $R_\mathrm{g}$ has an error of 0.5 Å. We note that the difference between the radii of gyration for the CYANA-based and RigiFlex based ensembles are well within this uncertainty. Therefore, it appears unlikely that additional small-angle scattering data would resolve the discrepancy between the two ensembles.

### CYANA multi-state modelling with only PRE restraints can reveal inter-RRM contacts

The ensemble structure determination strategy described so far requires great experimental effort for generating many spin-labelled double mutants for DEER measurements and substantial computational effort for RigiFlex computations including jack-knife resampling. We tested how much information can be obtained by a slim approach that uses only PRE restraints and generates the ensemble by faster CYANA modelling. Classical CYANA computations produced structures with high values of the target function and a large number of violated restraints, because these calculations attempt to find a single conformation that is consistent with all input restraints, rendering them inaccurate for dynamic ensembles. Consequently, we employed the novel multi-state CYANA calculation method, using PRE data as ambiguous restraints that do not need to be satisfied by each individual calculated state but only by the ensemble of states as a whole [21, 22].

**Figure 7.**
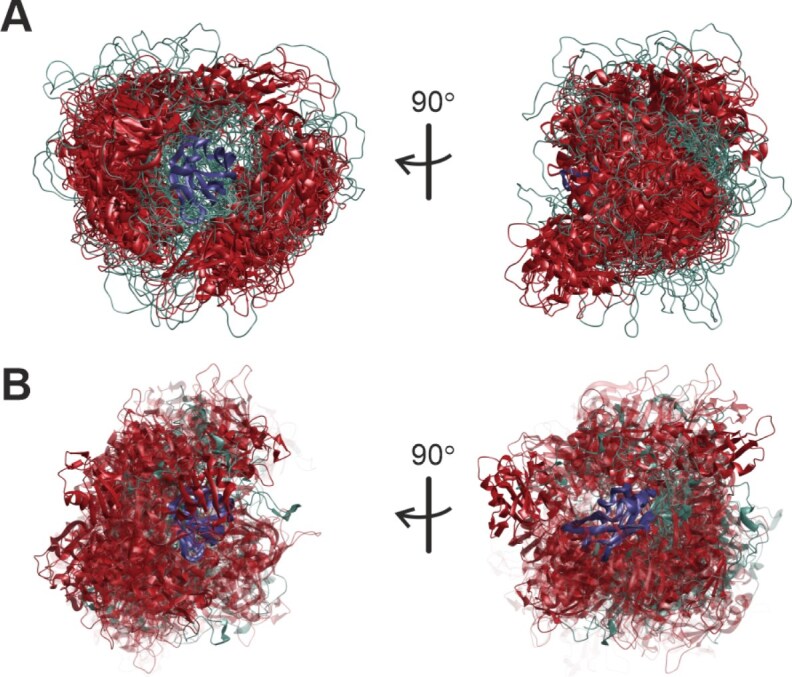
Comparison of ensembles obtained by (**A**) CYANA multi-state modelling (only PRE restraints) and (**B**) RigiFlex with jack-knife resampling and reweighing from the validation superensemble using both DEER EPR and PRE restraints. Conformers are superimposed upon RRM2 (residues 121–195, blue) with RRM1 displayed in red and the linker in cyan colour.

We systematically varied the number of states and found that the target function, number of violations, and maximum violation were largely converged with seven states, corresponding to an ensemble with 140 conformers. In this ensemble (Fig. 7A), the two RRMs occupy a restricted space close to each other, in agreement with the RigiFlex ensemble (Fig. 7B). Here, we superimposed conformers on RRM1, as this view better reveals the differences. Furthermore, we backcalculated the DEER distance distributions, which surprisingly exhibited a remarkable agreement with the experimental restraints (overlap deficiency $1-\bar{o} = 0.223$ and EMD of 5.4 Å), see Table 1 and Fig. 5, even though this ensemble contains no prior knowledge of the DEER data. This unexpected and reassuring result demonstrates efficacy of the multi-state CYANA calculation approach for our system, even though the more elaborate approach, which includes DEER data and ensemble reweighing, leads to substantially better fits of backcalculated PRE data and distance distributions. This result is remarkable as neither of our approaches utilizes electrostatic or other force-field information that could provide information on inter-domain interaction. Furthermore, the data from two independent experiments (PRE and DEER) demonstrate a high level of consistency with each other, although they are performed at different temperatures. Using both types of restraints, excludes some region of conformational space that is populated with only PRE restraints in the CYANA multi-state computation [RRM1 (red) on the right from RRM2 (blue) in the left panel of Fig. 7A] and conversely populates a small region of conformational space that is missing (RRM1 on the top left of RRM2 in the right panel of Fig. 7B).

Since PRE restraints are most sensitive to short distances, we conjectured that the CYANA multi-state ensemble should be informative on contacts between the RRMs. This expectation was borne out by contact analysis (Supplementary Fig. S12). We considered two residues as being in contact when at least one pair of atoms from the two residues had a distance of 3 Å or shorter. While the contact maps from the CYANA multi-state ensemble (Supplementary Fig. S12A) and from the reweighed RigiFlex superensemble (Supplementary Fig. S12C) differ in detail, they exhibit common features. Most of the residues that exhibit contacts to the other RRM in a substantial fraction of the conformer population do so in both ensembles. The residue that exhibits most contacts and the largest total percentage of contacts to the other RRM is Trp134 in both ensembles (Supplementary Fig. S12B and D). In general, the CYANA multi-state ensemble is enhanced in contacts, as the sole use of short-range PRE restraints biases the ensemble towards more compact conformers. This is also apparent by the backcalculated distance distributions C16 T169C, Y37C T169C, Y72C T169C, Y37C A107C, and A107C C148 (Fig. 4) that for the CYANA multi-state ensemble all feature distribution mass at shorter distances than is experimentally observed with DEER, which cannot detect distances shorter than about 15 Å.

### The interaction between the RRMs is weak and multi-valent, but not unspecific

The contact map and contact fractions reveal that many residues from both RMMs are in contact with residues from the respective other RRM in only part of the conformers. The most likely explanation is dynamic interaction between the two RRMs, where a multitude of weak interactions keeps the domains together. Yet, the contact maps from both ensembles drawn from different initial pools and informed by different restraint sets reveal common preferences.

**Figure 8.**
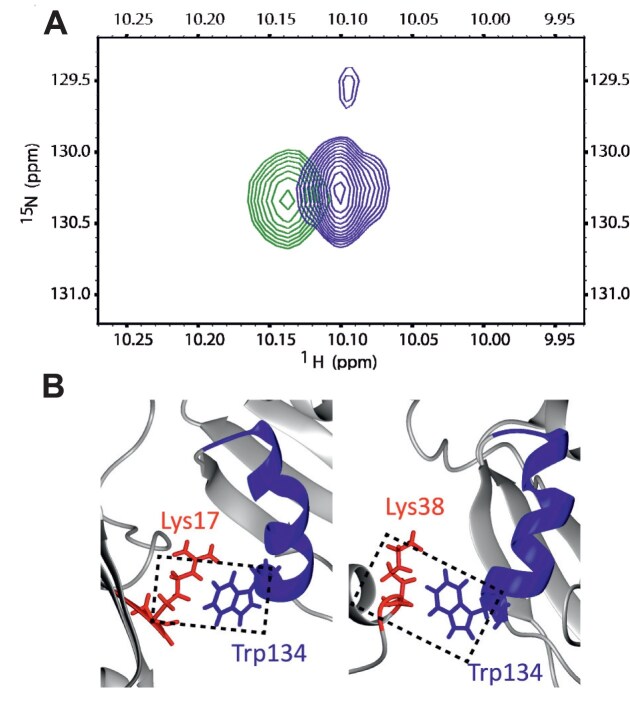
Transient contacts formed by Trp134 with RRM1 residues. (**A**) Chemical shift change for a Trp134 sidechain proton seen in the overlay of $^{1}$H-$^{15}$N HSQC spectra of SRSF1$_{\Delta \mathrm{RS}}$ (green) and the RRM2 construct (blue). (**B**) Interactions between Trp134 in RRM2 $\alpha _1$-helix and Lys residues in RRM1 observed in two conformers of the multi-state CYANA ensemble.

For instance, Trp134 in RRM2 contacts Lys17 in RRM1 in 4 out of the 140 conformers of the CYANA multi-state ensemble, corresponding to 2.9% of the conformers. The same applies to contacts between Trp134 and Lys38. Indeed, the sidechain N-H proton (H$^\epsilon$) of Trp134 exhibits a significant chemical-shift difference between spectra of isolated RRM2 and free SRSF1$_{\Delta \mathrm{RS}}$ (Fig. 8A). This behaviour is most likely explained by cation–$\pi$ interaction (Fig. 8B).

### AlphaFold3 predicts binding mode of RNA9, but not of RNA12

**Figure 9.**
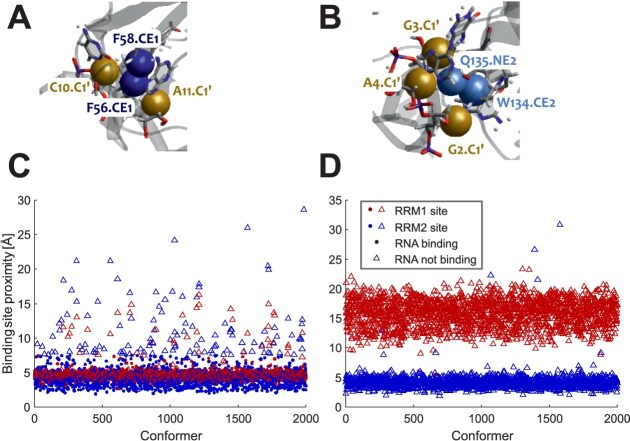
Prediction of RNA binding to RRM1 and RRM2 by AF3. (**A**) Binding of the CA motif to RRM1. (**B**) Binding of the GGA motif to RRM2. (**C**) Proximity of the CA motif to RRM1 and the GGA motif to RRM2 in 2000 AF3 models for the complex of SRSF1$_{\Delta \mathrm{RS}}$ with RNA9. Conformers are shown as dots if for both binding motifs at least one protein–nucleotide atom pairs is below the threshold distance of 7.5 Å. Otherwise, they are shown as open triangles. Red symbols correspond to CA/RRM1 binding and blue symbols to GGA/RRM2 binding, with darker shades for conformers below the binding threshold and lighter shades for those above the threshold. (**D**) Proximity of the CA motif to RRM1 and the GGA motif to RRM2 in 2000 AF3 models for the complex of SRSF1$_{\Delta \mathrm{RS}}$ with RNA12. Symbols and colours as in panel C.

AlphaFold is trained mainly against crystal structures. As very few of them involve binding of single-stranded flexible RNA regions to proteins, it is not clear whether AF3 can predict such binding. To address this question, we generated 2000 AF3 models each for the complexes of SRSF1$_{\Delta \mathrm{RS}}$ with RNA9 and RNA12 and tested whether they conform to the binding pattern detected recently by SELEX [14]. To this end, we considered distances between two protein atoms and two nucleotide atoms in the binding of CA to RRM1 (Fig. 9A) and between two protein atoms and three nucleotide atoms in the binding of GGA to RRM2 (Fig. 9B). For each AF3 model, we determined the shortest distance between one of the protein atoms and one of the nucleotide atoms. These distances are plotted in Fig. 9C and D for the RNA9 and RNA12 complex, respectively. For quantitative assessment, we consider binding prediction as successful if this shortest distance is below 7.5 Å.

With these provisions, AF3 successfully predicts binding of CA to RRM1 and GGA to RRM2 in 1242 out of the 2000 models in the RNA9 complex, but only in a single model in the RNA12 complex. We conclude that the initial AF3 conformer pool for the RNA9 complex can be used for ensemble modelling, whereas the pool for the RNA12 complex will not lead to a realistic ensemble model. We refrained from filtering the AF3 pool for the RNA9 complex for only the models with the correct binding pose. Instead, we performed ensemble reweighing on the complete pool to assess whether the EPR DEER and NMR PRE restraints discriminate against models with the wrong binding pose. In the reweighed ensemble, 3 out of 38 models still feature the wrong binding pose (Supplementary Fig. S13). They comprise a total weight of 8%, compared to the 37.9% of models with a wrong binding pose in the initial pool. Hence, the restraints do discriminate against the wrong pose but are not sufficient for excluding it entirely. Given the high affinity ($K_\mathrm{d} = 58$ nM) compared to the concentrations used in our experiments, it is unlikely that the restraint set is affected by a fraction of free proteins or protein where the RNA binds to only one RRM. We conclude that the SELEX restraints are required for arriving at a realistic ensemble model using initial AF3 ensembles. In the RigiFlex initial pool they are fulfilled by construction, as the RRM1/CA pair and the RRM2/GGA pair are treated as rigid bodies.

### Binding of RNA9 and RNA12 causes different shifts in the conformational distribution

We generated ensemble models of the RNA9 and RNA12 complexes of SRSF1$_{\Delta \mathrm{RS}}$ by reweighing initial AF3 and RigiFlex pools. In the case of RigiFlex, we also performed jackknife resampling and reweighing of the superensemble as well as reweighing against only the PRE restraints. Properties and fit quality of the ensembles are reported in Table 1 and the distance distribution fits are reported in Supplementary Figs S14 and S15.

#### Ensemble model for the complex of SRSF1$_{\Delta \mathrm{RS}}$ with RNA9

For the RNA9 complex, the EMD and $\chi ^2_\mathrm{PRE}$ are only marginally better for the RigiFlex ensemble compared to the AF3 ensemble. Further slight improvement is obtained with the reweighed superensemble. Again, we find that reweighing the RigiFlex pool against only PRE restraints worsens agreement with the distance distributions (EMD increase from 3.7 to 6.6 Å).

Agreement with the PRE restraints is slightly worse than in the case of free SRSF1$_{\Delta \mathrm{RS}}$, whereas EMD for the reweighed superensemble is higher by >70%. This can be traced back to probability density in the distance distribution of mutant C16 T169C at long distances (see Supplementary Fig. S14 and Supplementary Table S2), which is not reproduced by the ensemble. Such erroneous long-distance contributions in DEER distance distributions can arise when maximum dipolar evolution time is too short to allow for robust discrimination between modulation and background [30]. This might indeed be the case for the C16 T169C distance distribution (see Supplementary Fig. S5).

The experimental distance distribution between protein site C16 and the 5′-end of RNA9 (C16 U1 in Supplementary Fig. S14) is narrower than the backcalculated distribution for any ensemble. In this case, the experimental data is very reliable, with clear oscillations due to the narrow distribution showing up in the primary DEER data (Supplementary Fig. S5). We tentatively assign the discrepancy to a systematic error in rotamer library modelling for the 5′-label. The modelled linker distribution is probably too broad, which becomes apparent only for site pairs where the distribution of the backbone-to-backbone distances is narrow. The narrow experimental distance distribution for the site pair C16 U1 indicates very well-defined binding of the CA motif to RRM1. This in turn supports treatment of the CA motif together with RRM1 as a rigid body in the RigiFlex approach.

Jackknife resampling shows good agreement of backcalculated distance distributions for omitted restraints and high similarity of the individual validation ensembles to the reweighed superensemble (Supplementary Fig. S16 and Supplementary Table S2). Hence, the restraint set for the RNA9 complex is also consistent and sufficient. We note that the ensemble is more strongly restrained by both the binding of the RNA, which imposes a narrow distance distribution between the binding motifs in the tandem RRMs, and the seven additional distance distributions that involve RNA sites. The reweighed superensemble (total pool size 5596 conformers) is again very similar to the initial RigiFlex ensemble at $s = 0.9953$.

The restraint imposed on the ensemble by binding RNA9 with a two-nucleotide linker UU between the CA and GGA binding motifs applies also to the 1242 models in the AF3 initial pools that feature the correct binding pose. Accordingly, this initial pool already provides a reasonable fit to the experimental distances distribution (EMD of 4.7 Å compared to 5.8 Å for free SRSF1$_{\Delta \mathrm{RS}}$, see also Supplementary Fig. S13). Ensemble reweighing further improves agreement to an EMD of 2.0 Å, which is, however, still worse than the one obtained with the RigiFlex initial pool (1.5 Å). Likewise, $\chi ^2_\mathrm{PRE}$ is slightly better with the RigiFlex pool (6.477) than with the AF3 pool (6.711). The similarity between the RigiFlex and AF3 reweighed ensembles of 0.8942 is somewhat lower than for free SRSF1$_{\Delta \mathrm{RS}}$ (0.9045), although this difference is hardly significant. As in the case of free SRSF1$_{\Delta \mathrm{RS}}$, the difference between the ensembles is a restriction of the AF3 ensemble to a smaller region of conformation space (compare Supplementary Fig. S18A and B). It appears likely that the AF3 initial pool does not sample all conformations that are populated in solution.

Our ensemble can be compared to a previously described ensemble for a similar SRSF1 construct in complex with a similar RNA construct, visualized in the left panel in Fig. 5C of [15] . A visualization of our ensemble of 48 conformers obtained from the RigiFlex pool is shown in Supplementary Fig. S17 A in a similar viewing direction and with matching colour code. While some similarities are apparent, our example populates some regions in space that appear unpopulated in the earlier ensemble with only 8 conformers. It appears unlikely that these differences result from the slightly longer RNA construct (5′-UUUCAUUGGAUU-3′) instead of 5′-UCAUUGGAU-3′ used in our earlier study. We attribute these differences to our larger set of 272 PRE restraints from 6 labelling sites instead of 46 restraints from a single labelling site and to the 17 DEER distance distributions that provide complementary information on arrangements with large RRM separations in our case.

#### Ensemble model for the complex of SRSF1$_{\Delta \mathrm{RS}}$ with RNA12

In the case of RNA12, the initial RigiFlex pool fits the experimental distance distributions already quite well and the agreement after reweighing is similar to the case of RNA9 (Supplementary Fig. S15 and Table 1). Again, the larger EMD of 1.6 Å as compared to free SRSF1$_{\Delta \mathrm{RS}}$ (0.8 Å) is caused mainly by an erroneous long-distance contribution in the experimental distance distribution for site pair C16 T169C (see also Supplementary Table S3). Interestingly, there is again a site pair where the experimental distance distribution is narrower than all backcalculated distributions. In the RNA12 complex, this site pair is C148 U1. In contrast, the even narrower distribution for site pair C16 U12 is nicely fitted by the backcalculation from the reweighed RigiFlex ensemble. This supports our conjecture above that the discrepancy arises from rotamer library modelling of the 5′-label.

As in the case of the RNA9 complex, jackknife resampling results in quite good agreement of backcalculated distributions for omitted restraints with experimental distributions (Supplementary Fig. S19). Likewise, similarity of the validation ensembles with the reweighed superensemble (total pool size of 3881 conformers) is high (Supplementary Table S3). This restraint set is consistent and sufficient as well. The similarity of the reweighed superensemble to the initial RigiFlex ensemble is very high at 0.9982.

We also performed ensemble reweighing on the initial AF3 pool. In this case, the ensemble is known to not represent the conformation distribution in solution, since all but one conformer in the initial pool do not feature binding of the CA motif to RRM1. Indeed, the distance distributions backcalculated from the AF3 initial pool (yellow lines in Supplementary Fig. S15) are in stark disagreement with experiment. The pool is diverse enough for fitting some of the experimental distributions upon reweighing but insufficient to fit all of them. Substantial discrepancies remain for site pairs C16 T169C, Y72 T69C, C16 U12, and, unsurprisingly, C16 U1 close to the missed binding site. Similarity of the AF3 ensemble to the RigiFlex ensemble is comparatively poor at 0.8381. Yet, neither the fits of the distance distributions nor the similarity parameter are extremely poor. The AF3 ensemble is mainly excluded by not fitting the earlier SELEX experiments [14]. This finding indicates that distance distribution-based ensemble modelling requires high-quality experimental data, a sufficient number of restraints, and good agreement of all restraints with experiment. That said, the AF3 ensemble indeed occupies a similar region of conformation space as the RigiFlex ensemble, as is apparent from comparison of Supplementary Fig. S18C with D. The restraint set enforces a similar distribution of the spatial arrangement of the tandem RRMs despite the fact that the RNA is not properly bound to RRM1.

#### Comparison of reweighed RigiFlex superensemble models

We consider the reweighed RigiFlex superensemble models as the best models that we can currently provide for free SRSF1$_{\Delta \mathrm{RS}}$ and its complexes with RNA9 and RNA12. The change in the conformational ensembles upon RNA binding is surprisingly moderate (Table 3). The lowest similarity between the protein conformational ensembles is encountered between free SRSF1$_{\Delta \mathrm{RS}}$ and the complex with RNA9, which is still high at 0.9032. The ensemble for the complex with RNA12 is more similar to the one for free SRSF1$_{\Delta \mathrm{RS}}$ (0.9463) than is the one for the complex with RNA9 (0.9208). The ensemble of the complex with RNA9 has the lowest disorder parameter of $0.380 \pm 0.020$ (Table 1), followed by the complex with RNA12 ($0.408 \pm 0.028$). Unsurprisingly, the disorder parameter is largest for free SRSF1$_{\Delta \mathrm{RS}}$ ($0.486 \pm 0.020$). Binding of RNA to the tandem RRMs induces an incomplete disorder-to-order transition, as is already apparent from the distance distributions narrowing on average, but remaining much broader than expected for site pairs in the same IFR (Fig. 2) [74]. Changes in the radius of gyration are also surprisingly minor (Table 1). The protein somewhat expands from $R_\mathrm{g} = (20.8 \pm 0.5)$ Å for free SRSF1$_{\Delta \mathrm{RS}}$ to $(21.5 \pm 0.5)$ Å for the complex with RNA12 and $22.1 \pm 0.4$ Å for the complex with RNA9.

**Table 3.** Similarities of ensemble models for free SRSF1$_{\Delta \mathrm{RS}}$ and its RNA complexes

System	Free	With RNA9	With RNA12
Free	1	0.9032	0.9463
With RNA9	0.9032	1	0.9208
With RNA12	0.9463	0.9208	1

The high similarity between all three ensembles and the increase of $R_\mathrm{g}$ upon RNA binding indicate that the conformation ensemble of free SRSF1$_{\Delta \mathrm{RS}}$ is primed for RNA binding by weak interaction between the tandem RRMs. This is a key result of the current study. The chemical shift difference plot (Supplementary Fig. S2B) and the broad distribution of relative orientation and translation of the two RRMs suggest that this interaction is rather unspecific.

These ensembles can be compared as well to an ensemble described for a similar SRSF1 construct in complex with the same RNA construct [15], which had been originally introduced in [14]. The visualization of our ensemble derived from the RigiFlex pool by reweighing with all restraints in Supplementary Fig. S17B corresponds to the right panel in Fig. 5C of [15] . Our ensemble of 59 conformers obtained from the RigiFlex pool by reweighing with 325 PRE restraints from 6 labelling sites and 17 DEER distance distribution restraints differs substantially from the earlier ensemble with 8 conformers obtained with 41 restraints from the single labelling site E120C. The spin label conformation distribution for this labelling site is visualized in Supplementary Fig. S18C and D. Given the broad spatial distribution of the label and its location in a loop region that adds backbone flexibility, PRE restraints from a single labelling site may have been insufficient for determining the distribution of the relative arrangement of the two RRMs.

To obtain additional insight into the differences between the ensembles, we analysed distance root mean square deviation matrices for conformer pools that included all conformers in the reweighed superensemble of free SRSF1$_{\Delta \mathrm{RS}}$ and in the reweighed superensemble of either the RNA9 or the RNA12 complex. We then clustered these merged ensembles combining all conformers of the free superensemble with all conformers of either RNA-bound superensemble, aiming for about 12 conformers per cluster. The result of further analysis depends only weakly on this choice of mean cluster size. The clusters can be of three types, clusters containing conformers from both free SRSF1$_{\Delta \mathrm{RS}}$ and the RNA complex (mixed clusters), clusters containing conformers from only free SRSF1$_{\Delta \mathrm{RS}}$ (free protein clusters), or clusters containing conformers from only the RNA complex (RNA complex clusters). For the combined conformers from the free protein superensemble and the RNA9 complex superensemble, we find only mixed clusters (83.1% of the population by weight) and free protein clusters (16.9%). This phenomenon is similar to the notion of conformational selection, where suitable conformations for binding preexist. In the case of the combined conformers from the free protein superensemble and the RNA12 complex superensemble, we find only mixed clusters (88.3%) and RNA12 clusters (11.7%). The latter fraction excluding conformers from the free superensemble is similar to the notion of an induced fit, where conformation changes upon binding. Note however that in this picture RNA12 binding corresponds to a mix of conformational selection and induced fit. With our data, we have no way of studying the kinetics of binding that is usually associated with the concept of conformational selection and induced fit. This analysis reveals how flexibility of the SRSF1 tandem RRM arrangement conveyed by the linker allows for promiscuous RNA binding in order to combine versatility with efficiency. Such promiscuous binding is a prerequisite for a splicing factor that needs to process various RNAs and still be selective to some extent. This is another key result of this study.

**Figure 10.**
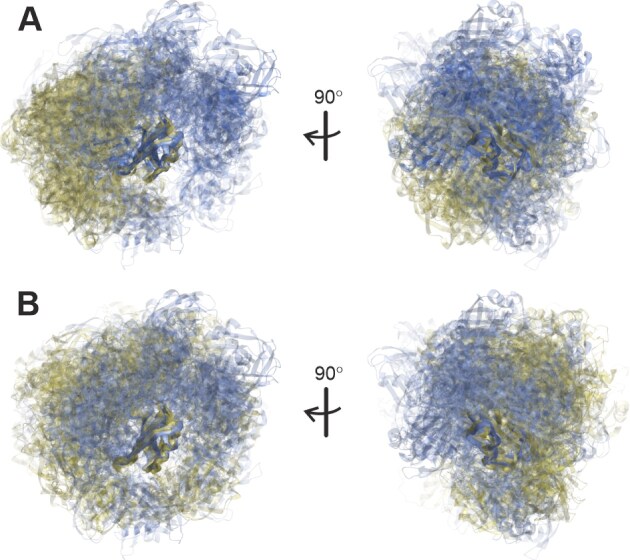
Shifts in the conformation ensemble upon binding of RNA9 and RNA12 by SRSF1$_{\Delta \mathrm{RS}}$. The reweighed RigiFlex superensemble of the free protein is shown in blue and the one of the RNA complexes in yellow. Transparency encodes conformer weight. (**A**) Binding of RNA9. (**B**) Binding of RNA12.

The result of this analysis is reinforced by visualization of the state transitions. Part of the space occupied by RRM2 (with respect to RRM1) in free SRSF1$_{\Delta \mathrm{RS}}$ (blue in Fig. 10) is vacated upon binding of RNA9 (gold). This space corresponds to the top right in the left panel and to the top front in the right panel. For binding of RNA12 such a depopulation of conformational space is less evident (Fig. 10B). The newly occupied space is on the left in the left panel and on the right front in the right panel.

This visualization also shows limitations of the analysis by clustering of conformers. The left panel in Fig. 10A clearly shows a higher density for the RNA9 complex (gold) on the left compared to the free protein (blue). The right panel in Fig. 10B shows higher density for the free protein than for the complex on the top left. Quantitative transition analysis that considers density of conformers in real space still needs to be developed.

## Discussion

Many RNA-binding proteins are promiscuous binders that still feature selectivity with respect to certain binding motifs. This is a prerequisite for a splicing factor such as SRSF1 that needs to process various RNAs but pose them in a specific way. Such behaviour requires some flexibility of the protein. One way to achieve this flexibility is the connection of RRMs by IDR linkers. The length and composition of such linkers might then provide another evolutionary handle for tuning RNA binding and conformation. With respect to disorder, three cases can be distinguished for RNA binding by flexibly linked tandem RRMs. In the first case, interaction between the two RRMs in the free state is strong and specific. There is no substantial arrangement disorder between the RRMs. The flexible linker allows for minor rearrangement of the binding sites to fit RNAs with slightly different steric requirements. In the second case, interaction between the RRMs is weak and unspecific or even absent, so that the free protein features arrangement disorder. Upon RNA binding, the linker folds or a defined interaction interface between the RRMs is formed. This corresponds to a disorder-to-order transition and is akin to folding upon binding. In the third case, the free protein also features arrangement disorder. However, RNA binding does not cause complete or near complete ordering. The RNA complex still exhibits substantial disorder. This is the case that we have encountered for SRSF1$_{\Delta \mathrm{RS}}$. The main evidence comes from broad site-to-site distance distributions for both the free protein and the complexes with RNA9 and RNA12. Narrow distributions are encountered only between a site in the RNA near a binding motif and sites in the RRM to which this motif binds (Supplementary Figs S14 and S15). Incomplete ordering is borne out by ensemble models and by their analysis in terms of the disorder parameter $\delta$.

Ensemble modelling in the presence of arrangement disorder between intrinsically folded domains is still in its early stages. To the best of our knowledge, such modelling was previously applied only once in the context of RNA binding. For the closed-to-open transition of the large subunit of the human U2 snRNP auxiliary factor (U2AF65) upon binding of 3′-splice-site-associated polypyrimidine tract RNA, an initial NMR study indicated narrrow, but distinct ensembles for the open and closed state [76]. A follow-up integrative study that included small-angle X-ray scattering (SAXS) data indicated a free-state ensemble that was much broader than the combined open- and closed-state NMR ensembles had indicated [77]. This free-state ensemble for U2AF65 is also much broader than the ensemble for free SRSF1$_{\Delta \mathrm{RS}}$.

Interpretation of such ensemble models suffers from difficulties in estimating their uncertainty [23, 24]. Three causes contribute to this difficulty. First, different ensemble models can fit the same data. If only mean-value restraints are available, even the width of the ensemble (extent of disorder) may be hard to estimate. We approached this problem by measuring distance distributions that directly inform on ensemble width. Second, conformational space for a protein with almost 200 residues, such as SRSF1$_{\Delta \mathrm{RS}}$, is huge, even if only the 30 residue linker connecting the RRMs is considered to be flexible. If a system is strongly disordered, one may not need to sample this space very densely, but one needs to sample fully the accessible space to avoid missing characteristic conformations. We approached this issue by comparing various sampling approaches. For free SRSF1$_{\Delta \mathrm{RS}}$, we applied unrestrained torsion angle dynamics in CYANA [35] and for all three systems we applied exhaustive search of rigid-body arrangement space by the Rigi module of MMMx [36]. Given the limited size of our initial pools, conformational sampling may still be sparse for the linker (residues 91–120) between the two RRMs. We note, however, that distance distributions involving site 107 near the middle of the linker are rather well reproduced for all three systems. While better sampling would be desirable, currently we do not have a better way to select from a conformer pool.

Third, it is hard to estimate whether a set of experimental restraints is sufficient and what constitutes good agreement with the experimental data while avoiding an overfit. The latter problem arises partially from unknown random or systematic errors in backcalculation of restraint data [78]. We approached this issue by balancing NMR PRE and EPR DEER restraint sets by normalization with respect to the best fit to restraints of only one type. This approach alleviates the problem, but does not fully solve it. This is apparent from $\chi ^2_\mathrm{PRE}$ values that greatly exceed 1, probably because of an oversimplified dynamics model in the backcalculation. It is also apparent from differences between the backcalculated and experimental distance distributions that exceed the 95% confidence interval of the experimental distributions obtained by a bootstrapping approach. This discrepancy appears to stem partially from limited sampling, as is seen by improvements between the initial RigiFlex ensembles and the reweighed superensembles. However, for the narrowest distributions in the RNA complexes, the remaining deviation exceeds sampling noise, pointing to limitations in rotamer library modelling of label conformation. All that said, the consistency between PRE restraints obtained at ambient temperature in liquid solution and of DEER distance distribution restraints obtained at 50 K for shock-frozen solutions is good in all three cases, which is encouraging.

We tested for consistency and sufficiency of the restraint sets by jackknife resampling. For all three systems, backcalculated distance distributions for the omitted restraints fitted experimental data reasonably well. Still, the EMD was substantially larger than for a fit with all restraints for most site pairs, indicating that even larger restraint sets could improve ensemble quality. However, the main current limitation may be sampling, as indicated by better consistency of ensembles obtained with the same sampling approach (RigiFlex) than between different sampling approaches (RigiFlex and CYANA) when using the same ensemble reweighing approach.

Finally we tested whether the knowledge on protein and protein-RNA complex structure encoded in AlphaFold can enhance ensemble modelling. Given that AlphaFold was trained on crystal structures and is firmly rooted in the Anfinsen paradigm by trying to predict a single conformer, it cannot be expected a priori that AlphaFold ensembles predict the conformation distribution in solution [37]. Indeed, our results indicate that AF3 computations with different random seeds for free SRSF1$_{\Delta \mathrm{RS}}$ and its complex with RNA9 do not fully cover the accessible conformation space. AF3 appears to overestimate interaction between the tandem RRMs. For RNA9, about 38% of the AF3 predictions fail to reproduce binding of either the CA motif to RRM1 or the GGA motif to RRM2. For RNA12, almost all AF3 models fail to correctly dock the CA motif to RRM1. Given the 58 nM affinity of the RNA constructs to SRSF1$_{\Delta \mathrm{RS}}$ in both cases, this is a sobering result. AF3 models for binding of flexible single-stranded RNA to proteins should not be accepted without experimental test. That said, the affinity of 58 nM is high in the context of our experiments, but not in a cellular context, where concentration of SRSF1-binding RNA is usually much lower. Our ensembles for the RNA complexes pertain only to the bound state that is expected to be in equilibrium with the free state of SRSF1.

We used a construct devoid of the RS domain of SRSF1 and containing a GB1 solubility tag. The solubility tag was considered in AF3-based modelling, but not with the other approaches for generating the initial conformer pools. Given the 15-residue long flexible N-terminal section of SRSF1 and the His-tag between the N terminus and the GB1 tag, we assume that a strong influence of the GB1 tag onto the conformer distribution is unlikely. This view is supported by the similarity in RRM arrangement and its disorder between AF3 modelling on the one side and RigiFlex and CYANA modelling on the other side. However, the AF3 models do feature a narrower conformational distribution and we cannot exclude that the presence of the GB1 tag contributes to this effect. We refrained from including the GB1 tag with the other approaches for initial pool generation, as this would require much more extensive sampling. Instead, we plan work on full-length SRSF1 without the solubility tag with a slightly adapted approach.

The RS domain influences binding of longer RNA and may also interact with the RRMs. Therefore, the ensembles in the current study are not necessarily characteristic for full-length wild-type SRSF1. We also note that solubility-enhancing mutation of tyrosine residues 37 and 72 may bias the ensemble with respect to the wild-type. Such bias could not be completely avoided in our spin labelling approach, as we use the same sites as labelling sites. We conjecture that conformation bias from removing the RS domain and mutating the two tyrosine residues would be stronger for free SRSF1 than for the RNA complexes. In the latter case, mutual arrangement of the RRMs and its disorder is governed by short linkers (2 or 5 nucleotides) between the two binding sites. The RNAs are too short for extensive interaction with the RS domain. However, the presence of some conformational bias for the RNA complexes cannot be completely excluded.

## Conclusion

Free SRSF1$_{\Delta \mathrm{RS}}$ is substantially disordered in solution. At the same time, the protein is much more compact than an ensemble model with unrestrained conformation of the flexible linker between the RRMs. This indicates weak, unspecific interaction between the two RRMs, which is in line with lower AlphaFold PAE between the RRMs than between the linker and each RRM. Disorder decreases only slightly upon binding RNA 5′-UCAUUGGAU-3′ (RNA9) and even less upon binding RNA 5′-UGGAUUUUUCAU-3′ (RNA12). In the former case, the majority of the conformational space occupied by the free protein remains occupied, whereas the population is redistributed. In the latter case, no substantial part of the conformation space occupied by the free protein appears to be depopulated, whereas population is redistributed as well and some part of conformation space is newly accessed. These observations provide some insight into binding of RNA with different directionality by the tandem RRMs of SRSF1. On a more general level, they show how promiscuous RNA-binding proteins can be primed for RNA binding while still allowing for binding rather different target sequences in order to combine versatility with efficiency.

Integrative ensemble modelling profits from distance distribution restraints that directly inform on ensemble width. Yet, even 10 distance distributions for the free protein and 17 distributions for each of the complexes leave some uncertainty about the occupied conformational space as is apparent from differences between ensembles for free SRSF1$_{\Delta \mathrm{RS}}$ that were obtained with different sampling approaches which all fit the experimental restraints reasonably well. The distance distribution restraints from EPR DEER and the NMR PRE restraints complement each other and are surprisingly consistent with each other, given that they are obtained on shock-frozen solutions at 50 K and on liquid solutions at ambient temperature, respectively. Backcalculation of PRE restraints probably involves some errors due to interconversion between conformers on the NMR time scale that is considered only approximately in the model. More sophisticated backcalculation of PREs that includes interconversion between conformers could not easily be reconciled with an ensemble reweighing approach. CYANA multi-state ensemble modelling with a slimmer approach requiring only PRE restraints reproduced distance distributions which were not included as input surprisingly well and provided information on interactions between RRMs.

Reasonably good fits of all restraints do not necessarily imply that the ensemble is realistic. This depends on sufficiency of the restraint set, which can be assessed by jackknife resampling. At the same time, this approach generates an ensemble of ensembles that can be used for obtaining uncertainty estimates on ensemble quantities, such as the radius of gyration or a disorder parameter. Similarity between the individual ensembles in this validation subensemble helps to assess sufficiency of the restraint set and the level on which the ensemble can be interpreted.

Many RNA-binding proteins are multi-functional and bind a large number of different RNA targets while maintaining selectivity to certain classes of targets. This implies flexibility, which may often be correlated with disorder of the free protein or even the RNA complexes in solution. Function of these proteins cannot be fully understood without understanding this balance between order and disorder. Our results with SRSF1 clearly show that depending on the bound RNA sequence and its specific positioning of the binding sites, either a redistribution of populations in the free ensemble can occur, or new conformations can be accessed. While AlphaFold predictions are valuable for generating high- resolution structural models for IFRs, such as the RRMs, they cannot be expected to sample the full conformational space that proteins access in solution. AlphaFold predictions can generate hypotheses on RNA binding. However, at the current stage experimental tests of such hypotheses appear to be mandatory.

Structural biology of RNA-binding proteins and their complexes within the Anfinsen paradigm, where a system is modelled by a single conformer, has provided much insight and will certainly provide more insight in the future. However, the state transitions of these proteins upon binding are not well approximated by transitions between two single conformers. For better understanding of these transitions, integrative ensemble structure modelling needs to be further developed and applied to more systems.

## Supplementary Material

gkag269_Supplemental_File

## Data Availability

Restraint files and ensemble structures have been deposited to Zenodo (DOI: 10.5281/zenodo.16950233).
