# Supplementary material for: Characterization of flexible RNA binding by tandem RNA recognition motifs through integrative ensemble modelling
Source: Nucleic Acids Res. 2026 Mar 31;54(6):gkag269. doi: 10.1093/nar/gkag269 (PMC13036500; doi:10.1093/nar/gkag269)
Supplement: gkag269_Supplemental_File [file gkag269_supplemental_file.pdf]

Supplementary Information:

Characterization of flexible RNA binding by tandem RNA recognition motifs through integrative ensemble modelling

Cristina K. X. Nguyen[1], Laura Esteban-Hofer[2], Fred F. Damberger[1], Maxim Yulikov[2], Peter Güntert[2,3,4], Laura Galazzo[2], Antoine Cléry[1], Gunnar Jeschke*[2], and Frédéric H.-T. Allain†[1]

[1]Department of Biology, ETH Zürich, Hönggerbergring 64, CH-8093 Zürich, Switzerland
[2]Department of Chemistry and Applied Biosciences, ETH Zürich, Vladimir-Prelog-Weg 2, CH-8093 Zürich, Switzerland
[3]Institute of Biophysical Chemistry, Goethe University, Max-von-Laue-Str. 9, 60438 Frankfurt am Main, Germany
[4]Department of Chemistry, Tokyo Metropolitan University, 1-1 Minami-Osawa, Hachioji, Tokyo 192-0397, Japan

January 22, 2026

Contents

S1 Additional Methods **3**
S1.1 Ensemble reweighing with earth mover's distance 3

S2 RigiFlex modelling steps **4**

S3 Additional Data **5**
S3.1 NMR data and AlphaFold3 pLDDT 5
S3.2 AlphaFold3 predicted aligned error 6
S3.3 NMR chemical shift analysis for free SRSF1$_{\Delta RS}$ 7

*Corresponding author
†Corresponding author

S3.4 Primary DEER data . . . 8
S3.5 Dependence of the A017C C148 distance distribution on pH . . . 9
S3.6 Distance distributions involving RNA sites . . . 10
S3.7 PRE data and fits for RNA complexes of SRSF1$_{\Delta\mathrm{RS}}$ involving RNA sites . . . 11
S3.8 Convergence of figures of merit in ensemble reweighing . . . 12
S3.9 Ramachandran plots for the inter-domain linker (residues 91-120) in free SRSF1$_{\Delta RS}$ . . . 13
S3.10 Comparison of the reweighed superensemble of free SRSF1$_{\Delta\mathrm{RS}}$ to the unrestrained CYANA pool . . . 14
S3.11 Jackknife resampling for free SRSF1$_{\Delta\mathrm{RS}}$ . . . 15
S3.12 Contact analysis between RRM1 and RRM2 . . . 16
S3.13 Binding of RNA9 in the reweighed AF3 ensemble . . . 17
S3.14 Fit of distance distributions by various ensembles of SRSF1$_{\Delta\mathrm{RS}}$ in complex with RNA9 . . . 18
S3.15 Fit of distance distributions by various ensembles of SRSF1$_{\Delta\mathrm{RS}}$ in complex with RNA12 . . . 19
S3.16 Jackknife resampling for the complex of SRSF1$_{\Delta\mathrm{RS}}$ with RNA9 . 20
S3.17 Ensembles of the two RNA complexes in a visualization that can be compared to [1] . . . 22
S3.18 Comparison of ensemble models for RNA complexes of SRSF1$_{\Delta\mathrm{RS}}$ 23
S3.19 Jackknife resampling for the complex of SRSF1$_{\Delta\mathrm{RS}}$ with RNA12 24

S4 Ensemble data format **26**

S1 Additional Methods

S1.1 Ensemble reweighing with earth mover's distance

In previous work, we fitted distance distributions by maximizing the overlap between the experimental and simulated distributions [2, 3, 4]. This figure of merit is more robust than the square norm of the fit residual with respect to sampling noise due to an insufficient number of conformers and with respect to "pearling" of experimental distributions that arises from the ill-posed nature of computing the distance distribution from experimental data. However, it is hard to relate to the uncertainty in predicting distance distributions between spin labels by the rotamer library approach [5], which is of the order of $2 \ldots 3$ Å [6]. Further, the mean overlap of distance distributions does not provide an intuitive understanding of the spatial uncertainty of the model. Finally, overlap of distance distributions does not have the mathematical properties of a distance metric.

Here we use the earth mover's distance (EMD) as a figure of merit that has the desired properties. The EMD is based on considering the two distributions as heaps of earth. In this picture, it answers the question how much earth needs to be transported over which distance in order to convert one distribution to the other. If the distance distributions are normalized so that total probability equals one for each of them, the EMD has units of a distance. It is non-negative and can thus be arithmetically averaged over different pairs of distributions, which in our case correspond to different pairs of spin label sites. The EMD is known synonymously as Wasserstein metric.

In general, computation of the EMD requires solution of a linear optimization problem. However, in our special case we deal with two 1D distributions $\mathbf{P}$ and $\mathbf{Q}$ that we can define as binned (discrete) distributions with the same bins of width δr (same distance axis). Computation of the EMD is then very efficient. We define a vector $\mathbf{W}$ of the same length as $\mathbf{P}$ and $\mathbf{Q}$ and index the elements by $k = 1 \ldots n$. The elements of $\mathbf{W}$ are given by

$$W_k = W_{k-1} + P_k - Q_k \ , \tag{1}$$

where we set $W_0 = 0$. The EMD is then given by

$$\mathrm{EMD} = \delta r \cdot \sum_{k=1}^{n} |W_k| \ . \tag{2}$$

In practice, we find that minimizing the EMD results in very similar back-calculated distance distributions and ensemble models as minimizing overlap deficiency as we did in earlier work. Accordingly, overlap deficiency increases only slightly when minimizing the EMD instead. The main difference is the larger weight given by EMD to larger distance shifts between non-overlapping parts of the distribution. We consider this as an additional desired feature. Values of the EMD smaller than ≈ 2 Å indicate a better fit than is expected from uncertainty of spin label rotamer library simulation. Values of the EMD larger

than ≈ 2 Å indicate additional uncertainty due to experimental error of the distance distributions or inconsistency of distance distribution restraints with other sets of restraints. Such inconsistency is signalled by loss of merit L that differs strongly from zero. Values $L > 1$ indicate serious inconsistency. Some inconsistency is expected, as different sets of experimental restraints are measured under different conditions and often for slightly different constructs. In our case, constructs differ by the number (one for PRE, two for distance distributions) and position of the spin labels. Measurement conditions differ because PRE is measured at ambient temperature in liquid buffer solution, while distance distributions are measured at a temperature of 50 K in glassy frozen solution containing a cryprotectant.

S2 RigiFlex modelling steps

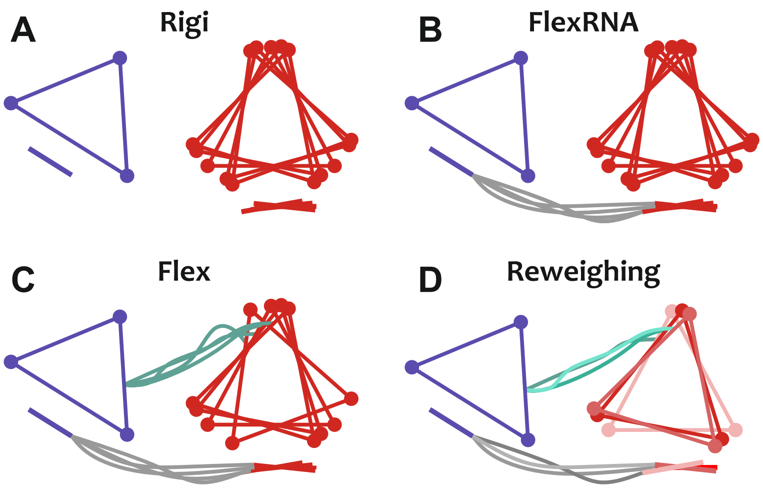

Figure S1: **Sections of the RigiFlex modelling pipeline for SRSF1$_{\Delta\mathrm{RS}}$.** (A) The Rigi section generates a pool of rigid-body arrangements of RRM1 (red triangle) with respect to RRM2 (blue triangle) by considering Gaussian distance distribution restraints between reference sites (filled circles). For RNA complexes, this arrangement includes the RNA binding motifs CA at RRM1 and GGA at RRM2 (straight lines). (B) For RNA complexes only, the FlexRNA section connects the RNA binding motifs by nucleotide linkers (grey) UU for RNA9 and nucleotides UUUUU for RNA12. (C) For all models, the Flex section generates the peptide linker (cyan) between RRM1 and RRM2 (residues 91-120). (D) The reweighing section, realized by the EnsembleFit module of MMMx [7], assigns weights to conformers (pictured by pale shades) and discards conformers with less than 1% of the maximum weight. This section is also used in ensemble modelling from the CYANA and AF3 pools.

S3 Additional Data

S3.1 NMR data and AlphaFold3 pLDDT

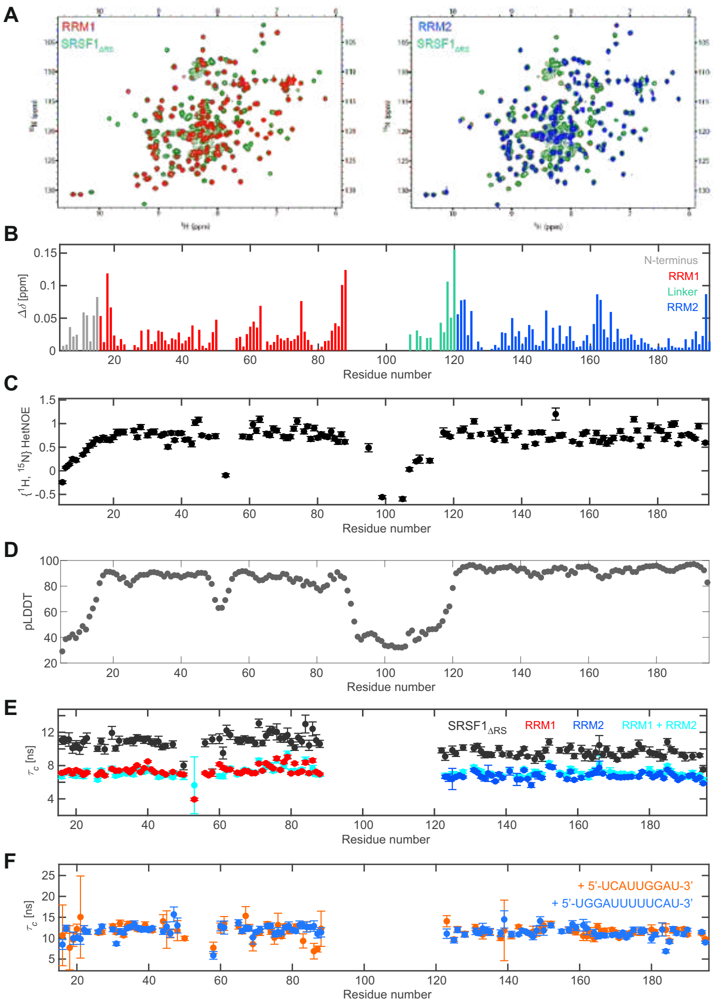

Figure S2: **NMR characterization of SRSF1$_{\Delta\mathrm{RS}}$.** (A) Overlay of ^{1}H-^{15}N HSQC spectra of SRSF1$_{\Delta\mathrm{RS}}$ (residue 1-196, green) and RRM1 (red) and RRM2 (blue). (B) Chemical shift differences of SRSF1$_{\Delta\mathrm{RS}}$ compared to single RRMs. Grey for the N-terminal region, red for RRM1, green for linker residues, and blue for RRM2. (C) HetNOE values. Error bars denote standard deviations. (D) AlphaFold pLDDT values. (E) Rotational correlation times of free versions of SRSF1 RRM1 (in red), RRM2 (in blue), RRM1+2 (in light blue) and SRSF1$_{\Delta\mathrm{RS}}$ (in black). (F) Rotational correlation times of SRSF1$_{\Delta\mathrm{RS}}$ bound to RNA9 (orange) and RNA12 (light blue).

S3.2 AlphaFold3 predicted aligned error

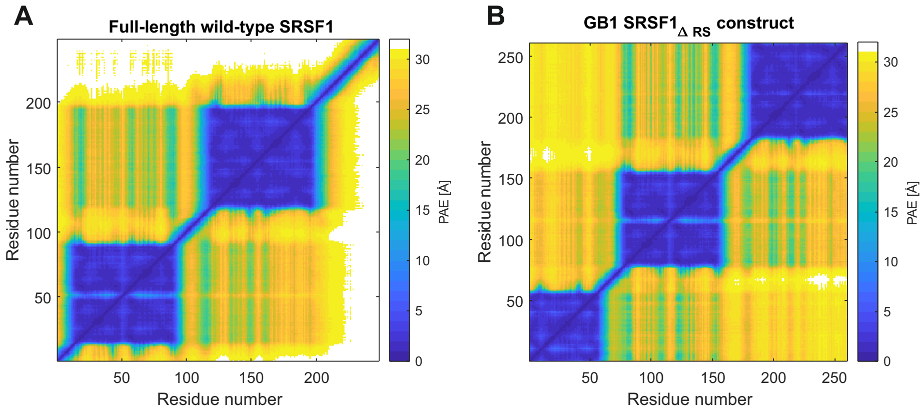

Figure S3: **AlphaFold3 predicted aligned error (PAE) matrices**. The matrix element (x, y) denotes the expected position error of the Cα atom of residue x when the predicted structure is superimposed with the "actual" structure at the backbone of residue y. In an ensemble picture, this is the ensemble aligned uncertainty, which is the 95% confidence interval of the postion of the Cα atom of residue x upon superposition of all conformers at residue y. White color indicates PAE at the capping value of 31 Å. (A) Full-length wild-type SRSF1 (UniProt Q07955). (B) Construct used in this work (see Figure 1 in main text) comprising a GB1 solubility tag and a HIS tag for purification and missing the RS domain (residues 197-248).

S3.3 NMR chemical shift analysis for free SRSF1$_{\Delta RS}$

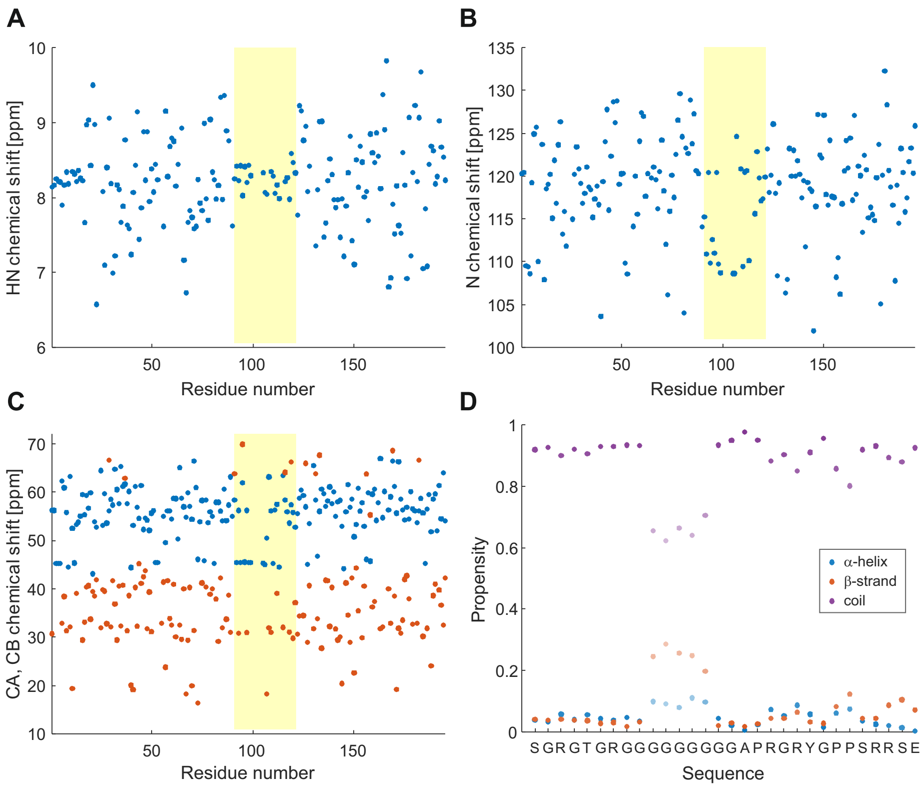

Figure S4: **NMR chemical shifts and secondary structure propensities for the inter-RRM linker in SRSF1$_{\Delta RS}$**. (A-C) Chemical shifts of (A) the HN, (B) the N, and (C) the Cα (blue) and Cβ (red) atoms. (D) TALOS-N secondary structure propensities for residues 91-120 in the inter-RRM linker. Confidence is encoded by transparency. The low-confidence predictions for a stretch of glycine residues with apparently lowered coil propensity are due to missing assignments for residues 100-104.

S3.4 Primary DEER data

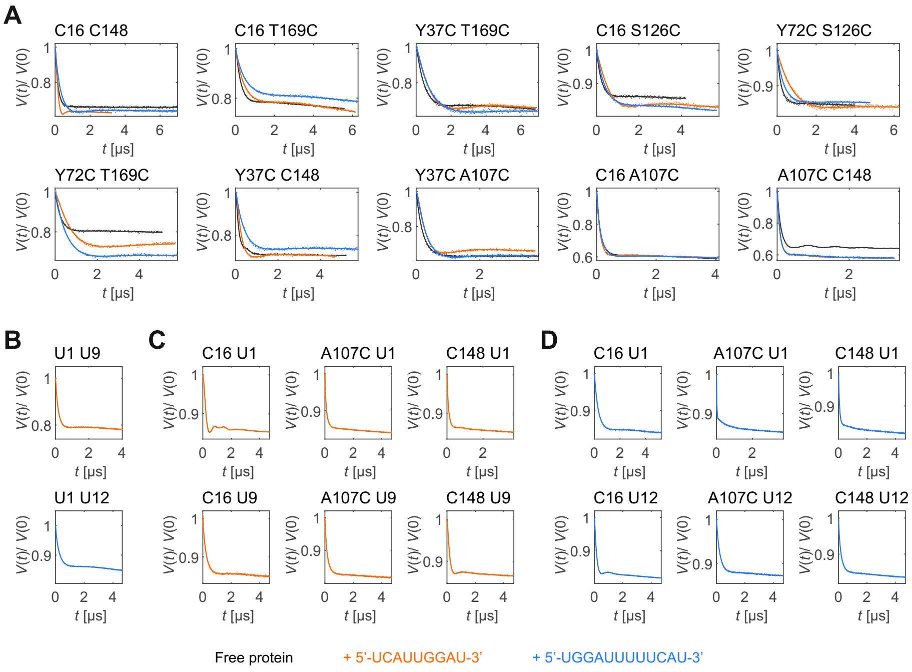

Figure S5: **Primary DEER data of SRSF1$_{\Delta RS}$.** Raw data (dots), median fits (solid lines) and corresponding 95% confidence intervals (shaded areas, derived from 1000 bootstrap samples) of DEER measurements acquired between (A) reference sites in the RRMs and in the RRM and A107C in the linker, (B) the 5'-end and the 3'-end of the RNA and (C-D) a site in the protein and a site in the RNA. Black denotes free protein, orange the complex with RNA9, and light blue the complex with RNA12.

S3.5 Dependence of the A017C C148 distance distribution on pH

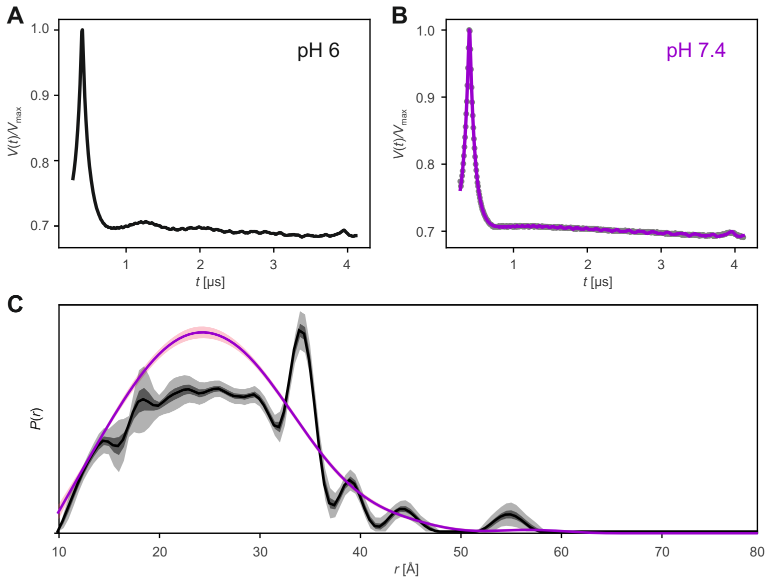

Figure S6: **DEER measurements of SRSF1$_{\Delta\mathrm{RS}}$ spin-labelled at A107C C148.** (A) Primary data for a measurement taken at pH 6. Clear oscillations are seen, indicating a narrow feature in the distance distribution. (B) Primary data for a measurement taken at pH 7.4. No oscillations are seen. (C) Distance distributions obtained from the data taken at pH 6.0 (black line and grey 95% confidence interval and taken at pH 7.4 (violet line and pale violet 95% confidence interval).

S3.6 Distance distributions involving RNA sites

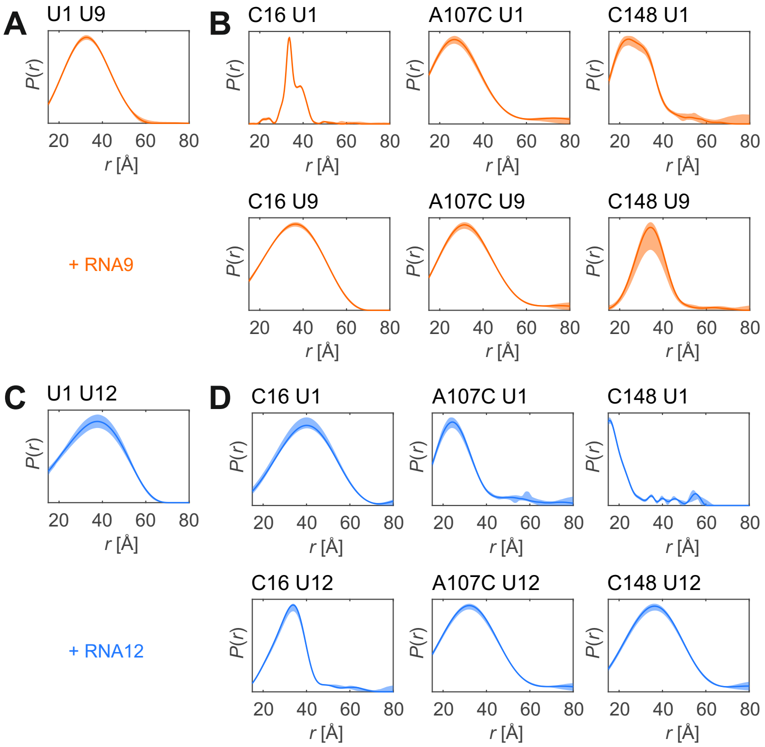

Figure S7: **Protein-RNA and RNA-RNA distance distributions.** Solid lines are the medians and shaded areas the 95% confidence intervals (from 1000 bootstrap samples). (A) RNA end-to-end distance distribution U1-U9 for SRSF1$_{\Delta\mathrm{RS}}$ in complex with RNA9. (B) Protein-RNA distance distributions for SRSF1$_{\Delta\mathrm{RS}}$ in complex with RNA9. (C) RNA end-to-end distance distribution U1-U12 for SRSF1$_{\Delta\mathrm{RS}}$ in complex with RNA12. (D) Protein-RNA distance distributions for SRSF1$_{\Delta\mathrm{RS}}$ in complex with RNA9.

S3.7 PRE data and fits for RNA complexes of SRSF1$_{\Delta RS}$ involving RNA sites

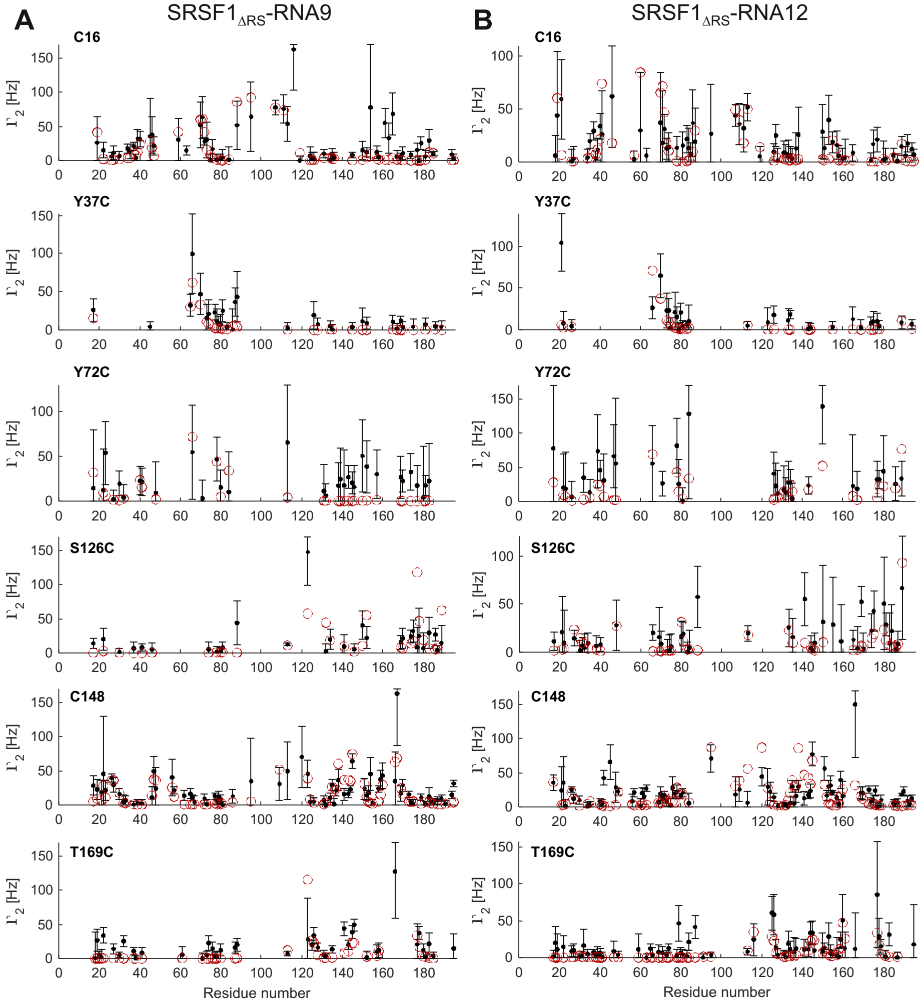

Figure S8: **NMR PRE data for RNA complexes of SRSF1$_{\Delta RS}$**. PRE rates are shown as black points with error bars. Backcalculated data (red open circles) are weighted means for the RigiFlex reweighed superensembles. Backcalculated data is not shown for residues where it was capped at 170 s^{-1}. (A) Complex with RNA9. (B) Complex with RNA12.

S3.8 Convergence of figures of merit in ensemble reweighing

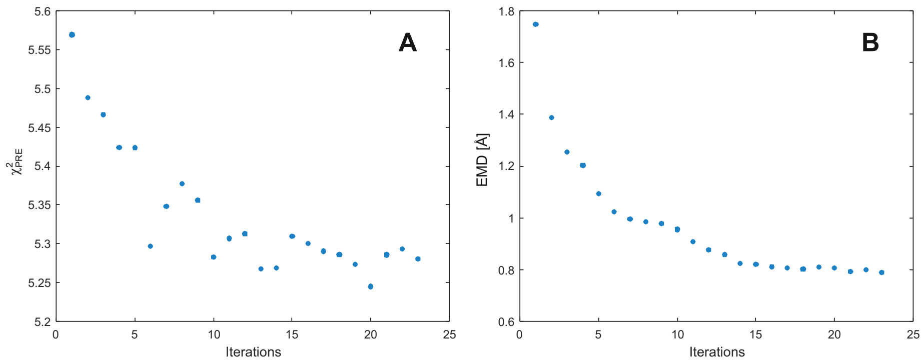

Figure S9: **Convergence of $\chi^2_{\mathbf{PRE}}$ and EMD during ensemble reweighing of the RigiFlex conformer pool for free SRSF1$_{\Delta RS}$.** In each iteration, new conformers were added from the pool to a total batch size of 100 conformers. (A) Convergence of χ^2_{PRE}. (B) Convergence of DEER distance distribution EMD.

S3.9 Ramachandran plots for the inter-domain linker (residues 91-120) in free SRSF1$_{\Delta RS}$

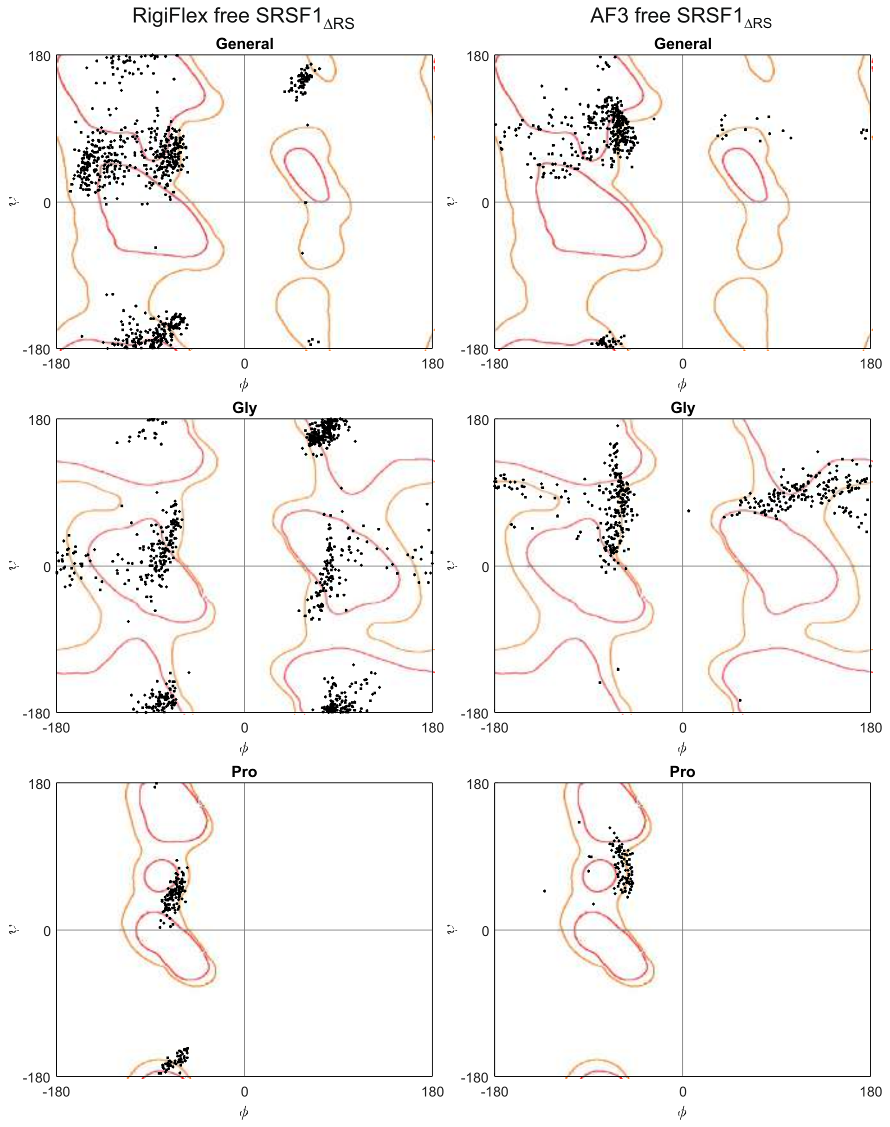

Figure S10: **Ramachandran plots for the inter-domain linker (residues 91-120) in free SRSF1$_{\Delta RS}$.** Data are shown separately for Pro, Gly, and all other residues (General). Red lines indicate the most favoured and orange lines the favoured regions. The left column refers to the ensemble derived from the RigiFlex pool by reweighing with all restraints and the right column to the ensemble derived from the AF3 pool.

S3.10 Comparison of the reweighed superensemble of free $SRSF1_{\Delta RS}$ to the unrestrained CYANA pool

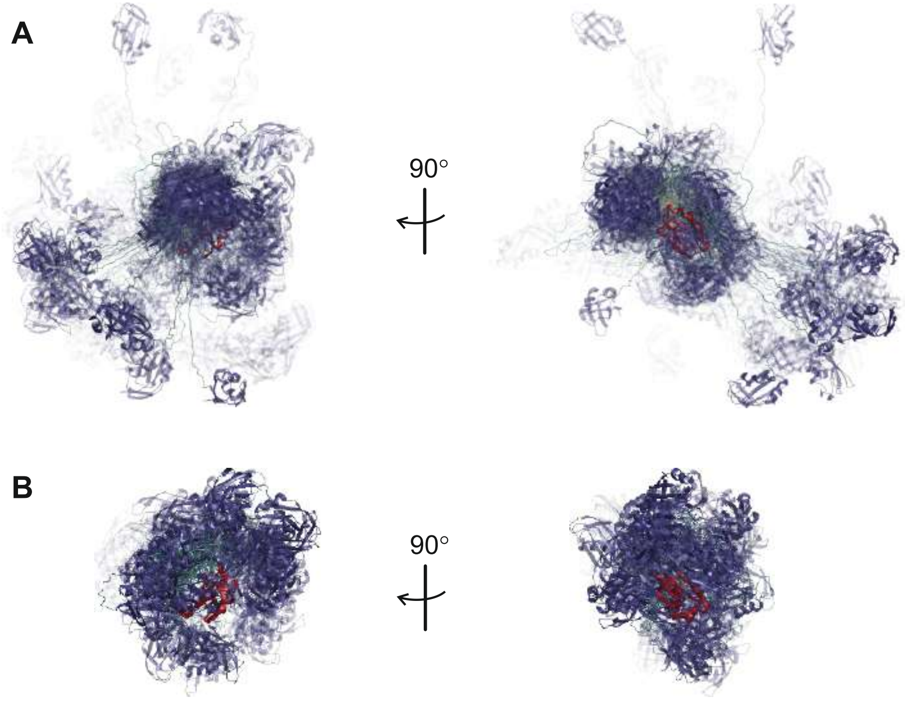

Figure S11: **Ribbon models of ensembles for free $SRSF1_{\Delta RS}$.** Conformers are superimposed on RRM1 (red) with transparency encoding weight. The linker is shown in cyan and RRM2 in blue. (A) Ensemble obtained by clustering the initial CYANA pool with 2000 conformers to 250 clusters. Each cluster is represented by the conformer with lowest total distance root mean square deviation to other conformers in the same cluster. Opaqueness is proportional to the number of conformers in this cluster. (B) Ensemble obtained by reweighing the conformers from the first RigiFlex ensemble and from the validation superensemble against all EPR DEER and NMR PRE restraints.

S3.11 Jackknife resampling for free SRSF1$_{\Delta RS}$

Validation of the restraint set by jackknife resampling was performed with smaller initial pools. In the following, we report the pool size, size of the reweighed and contracted ensembles, the earth mover's distance (EMD) between the backcalculated and experimental distribution for the omitted restraint, and the EMD for the same restraint in ensemble reweighing with all restraints (EMD_{full}) from an initial pool of similar size. We also report the similarity parameters $s_{superensemble}$ between the ensembles obtained by omitting one distance distribution restraint and the reweighed superensemble.

Table S1: Validation of the restraint set for free SRSF1$_{\Delta RS}$ by jackknife resampling. Similarity $s_{superensemble}$ for reweighing with all restraints from a RigiFlex pool with 312 conformers is 0.9879.

Omitted restraint	Pool Size	Conformers	EMD (Å)	EMD_{full} (Å)	$s_{superensemble}$
C16 S126C	313	33	4.8	1.3	0.9926
C16 C148	283	35	2.7	1.1	0.9937
C16 T169C	290	31	2.8	2.5	0.9925
Y37C C148	302	34	4.6	1.6	0.9785
Y37C T169C	278	38	7.0	1.1	0.9891
Y72C S126C	321	33	5.3	1.6	0.9844
Y72C T169C	290	38	3.1	1.7	0.9885
C16 A107C	379	40	5.0	0.7	0.9881
Y37C C107	302	41	4.3	1.2	0.9849
A107C C148	315	47	3.5	0.4	0.9925

S3.12 Contact analysis between RRM1 and RRM2

A residue pair (R_i, R_j) was assigned to be a contact pair if the atom-atom distance was below 3 Å for any pair where one atom was from residue R_i and the other one from residue R_j. This analysis was performed for all conformers in the ensemble and conformer weights w_c were added for all conformers where such contact occurred. We show parts of contact maps with residue R_i (abscissa) in RRM1 and residue R_j (ordinate) in RRM2 (Fig. SS12A,C). For each residue, we summed all total weights for contact residues in the other RRM. This data is shown in Supplementary Fig. S12B,D). In both cases, the maximum occurs for residue 134.

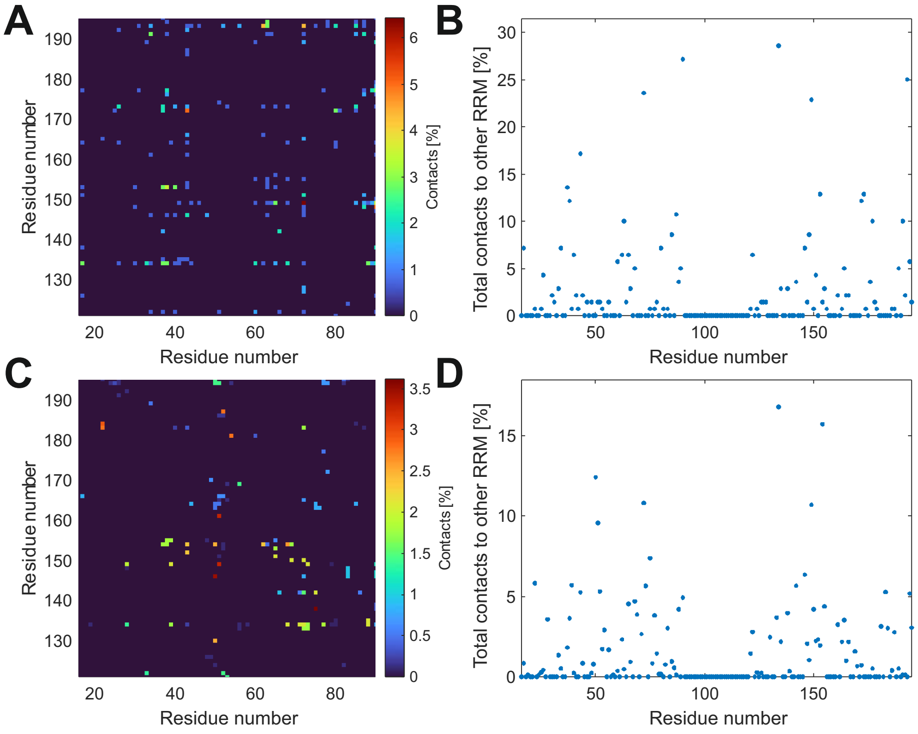

Figure S12: **Contacts between residues in different RRMs.** (A) Contact map for the CYANA Multi-state ensemble. (B) Total percentage of contacts to a residue in the other RRM for the CYANA multi-state ensemble. (C) Contact map for the reweighed RigiFlex superensemble. (D) Total percentage of contacts to a residue in the other RRM for the reweighed RigiFlex superensemble.

S3.13 Binding of RNA9 in the reweighed AF3 ensemble

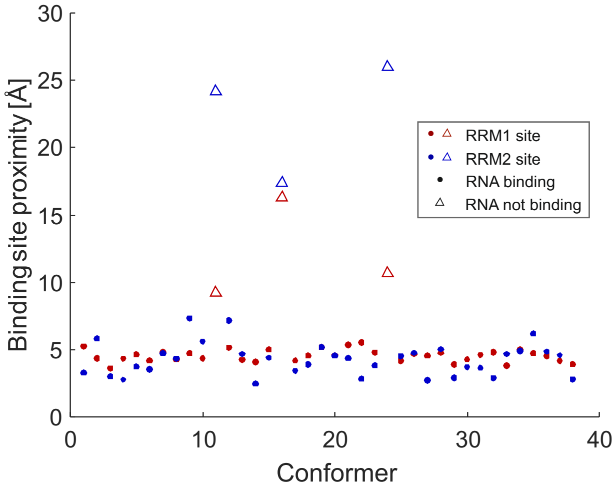

Figure S13: **Proximity of RNA binding motifs to RNA binding sites in RRM1 and RRM2 for the reweighed AF3 ensemble of SRSF1$_{\triangle RS}$ bound to RNA9.** Conformers are shown as dots if for both binding motifs at least one protein-nucleotide atom pairs is below the threshold distance of 7.5 Å. Otherwise, they are shown as open triangles. Red symbols correspond to CA/RRM1 binding and blue symbols to GGA/RRM2 binding. Three out of 38 conformers do not exhibit the proper binding pattern.

S3.14 Fit of distance distributions by various ensembles of SRSF1$_{\Delta RS}$ in complex with RNA9

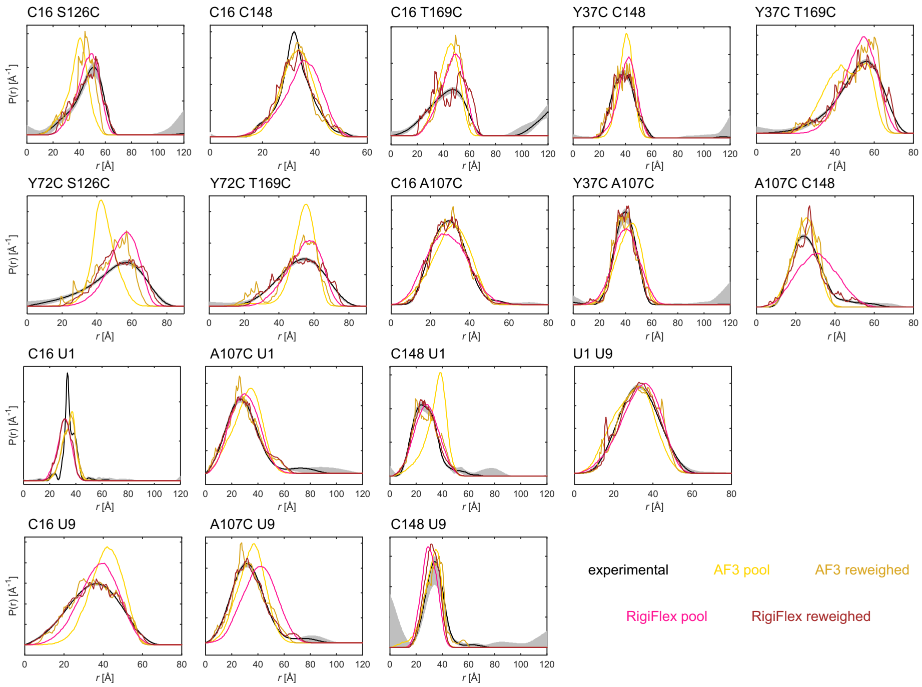

Figure S14: **Fit of distance distributions for SRSF1$_{\Delta RS}$ bound to RNA9.** Shown are the experimental distributions (black lines) with 95% confidence intervals (grey area) and backcalculated distributions from the initial AF3 pool (yellow) and RigiFlex pool (magenta). Backcalulated distributions after ensemble reweighing are displayed in darker shades (AF3 goldenrod, RigiFlex chocolate).

S3.15 Fit of distance distributions by various ensembles of SRSF1$_{\Delta\text{RS}}$ in complex with RNA12

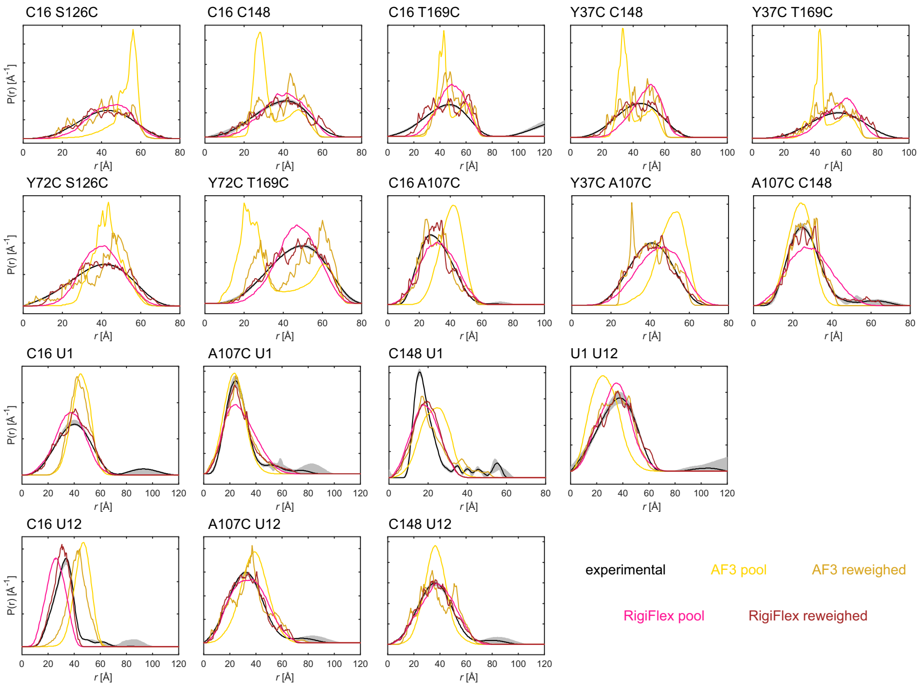

Figure S15: **Fit of distance distributions for SRSF1$_{\Delta\text{RS}}$ bound to RNA12.** Shown are the experimental distributions (black lines) with 95% confidence intervals (grey area) and backcalculated distributions from the initial AF3 pool (yellow) and RigiFlex pool (magenta). Backcalculated distributions after ensemble reweighing are displayed in darker shades (AF3 goldenrod, RigiFlex chocolate).

S3.16 Jackknife resampling for the complex of $SRSF1_{\Delta RS}$ with RNA9

Validation of the restraint set by jackknife resampling was performed with smaller initial pools. In the following, we report the pool size, size of the reweighed and contracted ensembles, the earth mover's distance (EMD) between the backcalculated and experimental distribution for the omitted restraint, and the EMD for the same restraint in ensemble reweighing with all restraints (EMD_{full}) from an initial pool of similar size. We also report the similarity parameters $s_{superensemble}$ between the ensembles obtained by omitting one distance distribution restraint and the reweighed superensemble.

Table S2: Validation of the restraint set for the complex of $SRSF1_{\Delta RS}$ with RNA9 by jackknife resampling. Similarity $s_{superensemble}$ for reweighing with all restraints from a RigiFlex pool with 292 conformers is 0.9943.

Omitted restraint	Pool Size	Conformers	EMD (Å)	EMD_{full} (Å)	$s_{superensemble}$
C16 S126C	274	45	3.1	1.4	0.9937
C16 C148	243	39	3.0	0.9	0.9876
C16 T169C	270	35	12.7	10.5	0.9867
Y37C C148	250	36	1.3	0.9	0.9921
Y37C T169C	274	38	1.2	0.9	0.9878
Y72C S126C	252	35	3.8	2.0	0.9956
Y72C T169C	274	35	1.7	0.6	0.9954
C16 A107C	257	38	0.9	0.5	0.9959
Y37C C107	302	31	1.8	0.6	0.9819
A107C C148	315	29	3.0	1.1	0.9914
C16 5'	248	42	5.2	3.8	0.9872
C16 3'	257	31	2.3	0.5	0.9928
A107C 5'	247	32	4.5	1.0	0.9903
A107C 3'	268	36	2.5	0.7	0.9914
C148 5'	208	32	3.3	2.1	0.9921
C148 3'	257	30	3.1	2.7	0.9878
5' 3'	260	34	2.3	0.4	0.9885

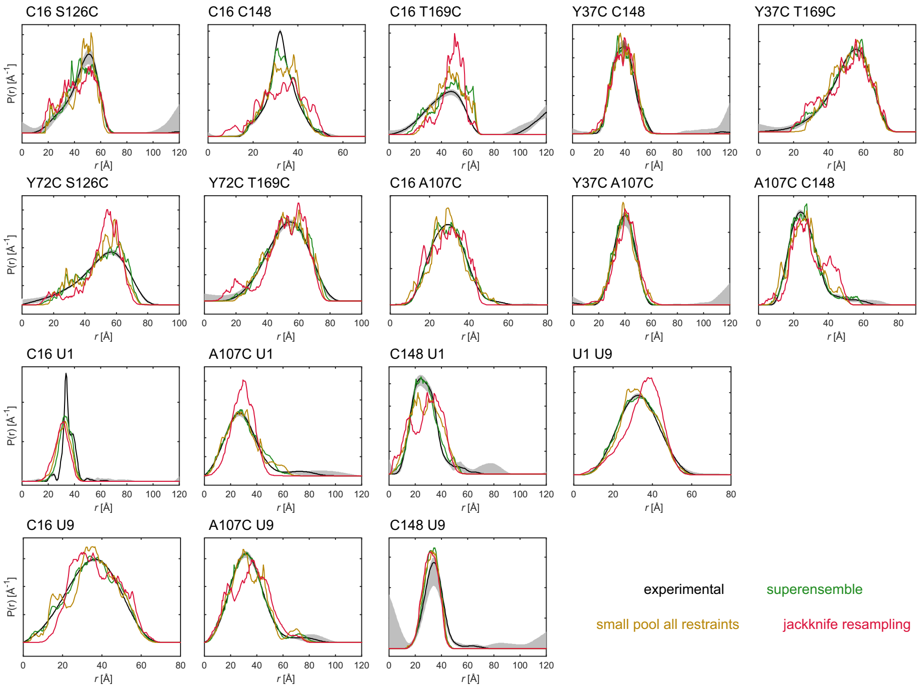

Figure S16: **Distance distribution fits in jackknife resampling for the complex of SRSF1$_{\Delta\mathrm{RS}}$ with RNA9.** Shown are the experimental distributions (black lines) with 95% confidence intervals (grey area) and backcalculated distributions from the reweighed superensemble (green), the ensemble obtained by reweighing against all restraints from a RigiFlex pool with 292 conformers (goldenrod), and by reweighing a RigiFlex pool computed without this restraint and omitting the restraint also in reweighing (magenta).

S3.17 Ensembles of the two RNA complexes in a visualization that can be compared to [1]

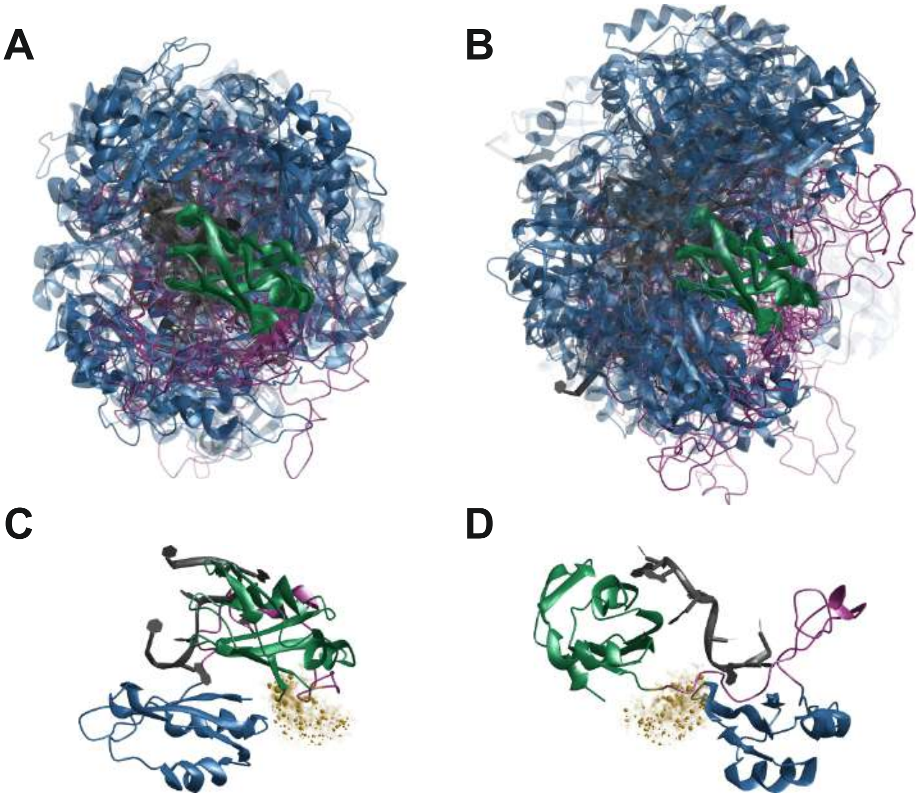

Figure S17: **Ensemble and spin label visualizations for comparison to earlier work.** Shown are cartoon plots in the approximate viewing direction and with a colour code corresponding approximately to Fig. 5C in [1]. All conformers are superimposed on RRM2 (green) and conformer weight is transparency-encoded. RRM1 is shown in blue, the linker in magenta, and RNA in grey. The spin label is visualized in goldenrod. (A) Complex of SRSF1$_{\Delta RS}$ with RNA9 (5'-UCAUUGGAU-3') [8] similar to construct uuuCAuuGGAu in [1]. (B) Complex with RNA12 (5'-UGGAUUUUUCAU-3') [8], which is the same as uGGAuuuuuCAu used in [1]. (C) Visualization of spin label conformation distribution as predicted by MMMx using the first conformer in our reweighed superensemble (goldenrod) at labelling site E120C in the same viewing direction (x) as for panels A,B. Spheres denote the location of the electron spin for different rotamers and sphere volume corresponds to predicted rotamer population. (D) Visualization of spin label conformation distribution at labelling site E120C in viewing direction y.

S3.18 Comparison of ensemble models for RNA complexes of SRSF1$_{\Delta RS}$

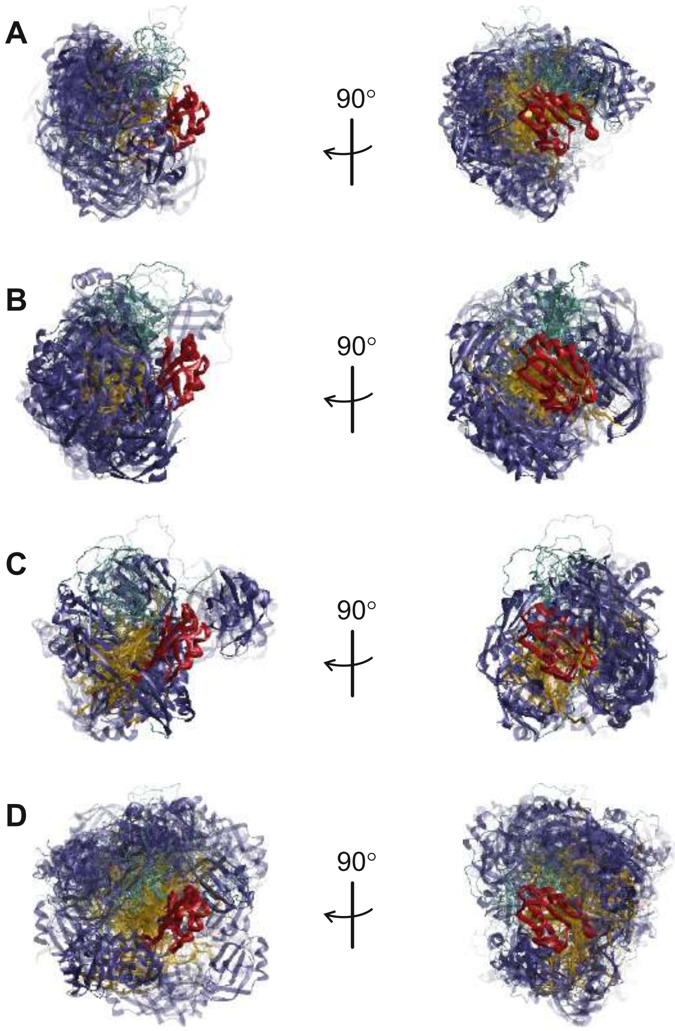

Figure S18: **Ribbon models of reweighed ensembles for RNA complexes of SRSF1$_{\Delta RS}$.** Conformers are superimposed on RRM1 (red) with transparency encoding weight. The linker is shown in cyan, RRM2 in blue, and the RNA in gold. All ensembles were fitted integratively to EPR DEER and NMR PRE restraints. (A) Ensemble for the complex with RNA9 obtained from the AF3 pool. (B) Ensemble for the complex with RNA9 obtained from the RigiFlex pool pool. (C) Ensemble for the complex with RNA12 obtained from the AF3 pool. (D) Ensemble for the complex with RNA12 obtained from the RigiFlex pool.

S3.19 Jackknife resampling for the complex of SRSF1$_{\Delta RS}$ with RNA12

Validation of the restraint set by jackknife resampling was performed with smaller initial pools. In the following, we report the pool size, size of the reweighed and contracted ensembles, the earth mover's distance (EMD) between the backcalculated and experimental distribution for the omitted restraint, and the EMD for the same restraint in ensemble reweighing with all restraints (EMD$_{\text{full}}$) from an initial pool of similar size. We also report the similarity parameters $s_{\text{superensemble}}$ between the ensembles obtained by omitting one distance distribution restraint and the reweighed superensemble.

Table S3: Validation of the restraint set for the complex of SRSF1$_{\Delta RS}$ with RNA12 by jackknife resampling. Similarity $s_{\text{superensemble}}$ for reweighing with all restraints from a RigiFlex pool with 136 conformers is 0.9802.

Omitted restraint	Pool Size	Conformers	EMD (Å)	EMD$_{\text{full}}$ (Å)	$s_{\text{superensemble}}$
C16 S126C	119	33	3.0	0.7	0.9788
C16 C148	104	29	1.8	1.0	0.9872
C16 T169C	137	28	10.0	9.4	0.9903
Y37C C148	138	34	4.3	2.4	0.9763
Y37C T169C	113	32	4.4	4.6	0.9893
Y72C S126C	80	26	3.4	2.7	0.9927
Y72C T169C	121	28	2.5	1.4	0.9637
C16 A107C	157	34	1.6	0.9	0.9896
Y37C C107	302	33	2.7	0.7	0.9896
A107C C148	315	29	3.0	1.7	0.9876
C16 5'	139	22	4.7	2.4	0.9698
C16 3'	143	30	4.5	3.4	0.9825
A107C 5'	162	34	3.3	1.9	0.9887
A107C 3'	172	33	3.6	0.9	0.9905
C148 5'	150	35	6.1	6.6	0.9825
C148 3'	145	31	4.1	1.5	0.9767
5' 3'	142	33	3.8	1.6	0.9858

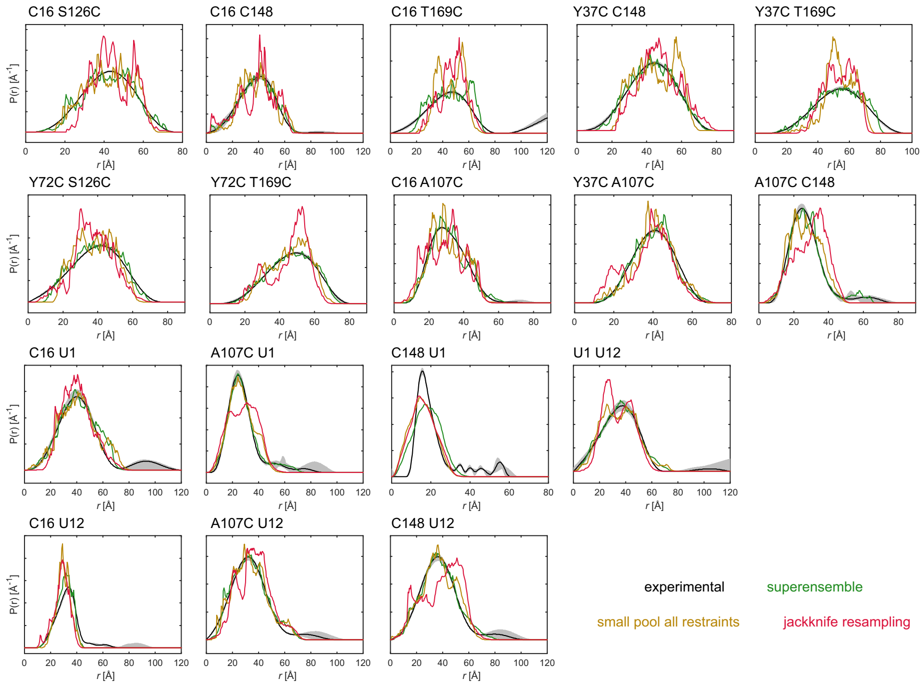

Figure S19: **Distance distribution fits in jackknife resampling for the complex of SRSF1$_{\Delta\mathrm{RS}}$ with RNA12.** Shown are the experimental distributions (black lines) with 95% confidence intervals (grey area) and backcalculated distributions from the reweighed superensemble (green), the ensemble obtained by reweighing against all restraints from a RigiFlex pool with 292 conformers (goldenrod), and by reweighing a RigiFlex pool computed without this restraint and omitting the restraint also in reweighing (magenta).

S4 Ensemble data format

Each ensemble listed in Table 1 of the main text is stored on Zenodo (DOI: 10.5281/zenodo.16950233) in a ZIP file. The ZIP file contains PDB files of all conformers in the ensemble as well as a list of these files with corresponding weights (extension .ens).

References

[1] Naiduwadura Ivon Upekala De Silva, Talia Fargason, Zihan Zhang, Ting Wang, and Jun Zhang. Inter-domain flexibility of human Ser/Arg-rich splicing factor 1 allows variable spacer length in cognate RNA's bipartite motifs. *Biochemistry*, 61(24):2922–2932, 2022.

[2] Gunnar Jeschke. MMM: A toolbox for integrative structure modeling. *Protein Sci.*, 27(1):76–85, 2018.

[3] Irina Ritsch, Laura Esteban-Hofer, Elisabeth Lehmann, Leonidas Emmanouilidis, Maxim Yulikov, Frédéric H-T Allain, and Gunnar Jeschke. Characterization of weak protein domain structure by spin-label distance distributions. *Front. Mol. Biosci.*, 8:636599, 2021.

[4] Georg Dorn, Christoph Gmeiner, Tebbe de Vries, Emil Dedic, Mihajlo Novakovic, Fred F Damberger, Christophe Maris, Esteban Finol, Chris P Sarnowski, Joachim Kohlbrecher, Timothy J Welsh, Sreenath Bolisetty, Raffaele Mezzenga, Ruedi Aebersold, Alexander Leitner, Maxim Yulikov, Gunnar Jeschke, and Frédéric H-T Allain. Integrative solution structure of PTBP1-IRES complex reveals strong compaction and ordering with residual conformational flexibility. *Nat. Commun.*, 14(1):6429, 2023.

[5] Yevhen Polyhach, Enrica Bordignon, and Gunnar Jeschke. Rotamer libraries of spin labelled cysteines for protein studies. *Phys. Chem. Chem. Phys.*, 13(6):2356–2366, 2011.

[6] Gunnar Jeschke. Conformational dynamics and distribution of nitroxide spin labels. *Prog. Nucl. Magn. Reson. Spectrosc.*, 72:42–60, 2013.

[7] Gunnar Jeschke and Laura Esteban-Hofer. Integrative ensemble modeling of proteins and their complexes with distance distribution restraints. *Methods Enzymol.*, 666:145–169, 2022.

[8] Antoine Cléry, Miroslav Krepl, Cristina K X Nguyen, Ahmed Moursy, Hadi Jorjani, Maria Katsantoni, Michal Okoniewski, Nitish Mittal, Mihaela Zavolan, Jiri Sponer, and Frédéric H-T Allain. Structure of SRSF1 RRM1 bound to RNA reveals an unexpected bimodal mode of interaction and explains its involvement in SMN1 exon7 splicing. *Nat. Commun.*, 12(1):428, 2021.
